# A review of self-supervised imaging phenotypes for genome-wide association studies

**DOI:** 10.1038/s42003-026-11199-4

**Published:** 2026-10-23

**Authors:** Khush Patel, Xingzhong Zhao, Ziqian Xie, Degui Zhi

**Affiliations:** 1 J.C. Walter Jr. Transplant Center, Department of Surgery, Houston Methodist Hospital, Houston, TX USA; 2 McWilliams School of Biomedical Informatics, University of Texas Health Science Center at Houston, Houston, TX USA; 3 Department of Biomedical Informatics and Data Science, Yale University School of Medicine, New Haven, CT USA

**Keywords:** Machine learning, Image processing, Genome informatics, Computational neuroscience

## Abstract

Biobank-scale imaging-genomic cohorts enable genome-wide association studies (GWASs) of organ structure and function. Traditional approaches rely on expert-defined image-derived phenotypes (IDPs), which capture only a fraction of the biological variation in high-content images. Powered by artificial intelligence, self-supervised learning (SSL) learns representations directly from intrinsic image structures, creating scalable, data-driven traits. Here, we review the technical frameworks and challenges of SSL-based imaging genetics and their applications in brain MRI, cardiac MRI, and retinal imaging. Early studies suggest that these data-driven approaches can complement traditional IDPs and may expand the phenotypic space available for discovery of heritable imaging traits, although broader validation across cohorts, ancestries, scanners, and imaging protocols remains needed.

## Introduction

The availability of large-scale biobanks with imaging and genomic data has enabled studies of how genetic variation influences organ morphology and function through genome-wide association studies (GWASs). Imaging GWASs have shown that genetic variants contribute to structural and functional variation in organs such as the brain^1^, heart^2^, and eye^3^, thereby improving understanding of disease pathways^4,5^.

Imaging genetics has evolved substantially over the past two decades. Early studies often focused on candidate genes, linking variants such as *APOE* ε4, 5-HTTLPR, and *BDNF* Val66Met to brain MRI phenotypes, including measures of brain structure and task-related activation^6–8^. However, many of these associations were based on small samples and showed inflated effect sizes; subsequent meta-analytic and replication studies found that some candidate-gene effects were smaller, heterogeneous, or difficult to replicate consistently^9,10^. This replication crisis highlighted the highly polygenic architecture of imaging traits and drove a shift toward genome-wide analyses in larger, better-powered datasets. Large collaborative efforts such as the ENIGMA and CHARGE consortia helped establish the first robustly replicated loci for imaging phenotypes by harmonizing imaging and genomic data across multiple cohorts^11–13^. More recently, population-scale biobanks with integrated imaging and genetic data have expanded imaging-genetic discovery across multiple organ systems, including cardiac, retinal, abdominal, and whole-body imaging phenotypes.

Imaging-genetic resources can be broadly categorized into population-level biobanks, cohort studies, and consortia. Population-level biobanks recruit broadly from general populations, providing the sample size needed for GWAS. The UK Biobank (UKBB) is one of the largest and most prominent resources, with ~500,000 participants with genomic data and ~100,000 who have undergone multimodal imaging across brain, cardiac, abdominal, and retinal modalities^14–17^. Similarly, the German National Cohort (NAKO) has enrolled ~200,000 adults, including a whole-body magnetic resonance imaging (MRI) sub-cohort of ~30,000 participants^18,19^. With whole-genome sequencing currently underway, these resources will enable future large-scale imaging-genetics studies. The Qatar Biobank targets up to 60,000 nationals and long-term residents, combining genetic data with whole-body and brain MRI to provide a valuable resource of a Middle Eastern population^20^. In contrast, specialized cohorts and consortia target specific diseases. Representative examples include the Alzheimer’s Disease Neuroimaging Initiative (ADNI), which follows ~2400 participants with genomic data and longitudinal brain imaging^21,22^; the Adolescent Brain Cognitive Development (ABCD) Study, a pediatric neuroimaging and genomic resource with ~11,880 children^23,24^; the Multi-Ethnic Study of Atherosclerosis (MESA)^25^, which provides cardiac and vascular imaging across diverse ethnic groups along with genomic data; the Rotterdam Study with brain and retinal imaging along with genomic data^26^; and the Framingham Heart Study with longitudinal cardiovascular and genomic data^27,28^.

A key component of imaging GWAS is the derivation of heritable, interpretable, and reproducible phenotypes from medical images. Traditional approaches rely on expert-defined image-derived phenotypes (IDPs), typically based on anatomical segmentations and hand-crafted measurements obtained either manually or through automated software tools^1,29–31^. While powerful, these strategies may introduce bias from human annotation and assumptions embedded in segmentation software^32–34^, require labor-intensive labeling and may be constrained by current clinical knowledge of anatomy-function relationships. As a result, they may capture only a fraction of the information encoded in high-dimensional imaging data^35^. These limitations highlight the need for new approaches that can scale to biobank-level datasets while uncovering novel, biologically meaningful features beyond the scope of traditional IDPs. The biological challenge is therefore to convert complex medical images into scalable, reproducible, and heritable quantitative traits that preserve meaningful inter-individual variation and can be linked to genetic variation.

Self-supervised learning (SSL) models have emerged as an alternative approach for deriving phenotypes from medical images^36–41^. The goal of SSL is to learn a latent representation capturing the intrinsic structure of the input data modality. Instead of relying on human-labeled data in typical supervised learning, SSL supplies the prediction targets for each training example and trains itself using typical supervised losses such as input reconstruction, future prediction, or cross-encoder branch alignment^42,43^. In imaging genetics, SSL serves as a representation-learning framework for deriving quantitative imaging phenotypes from high-dimensional imaging data. These learned representations can then be used as imaging traits in downstream GWAS to identify genetic variants associated with imaging-derived phenotypic variation. For representation learning for medical images, SSL models enable label-free feature extraction directly from minimally processed images, without reliance on expert-defined labels^42^. Here, expert-defined refers to imaging phenotypes specified a priori by domain experts, such as radiologists, neurologists, cardiologists, ophthalmologists, or imaging scientists, based on established anatomical structures or clinical measurements. Examples include hippocampal volume, ventricular volume, left ventricular ejection fraction, retinal layer thickness, and vessel caliber. By learning representations via reconstruction or contrastive objectives, SSL can capture subtle, distributed anatomical patterns that may not be fully summarized by conventional phenotypes derived from predefined anatomical structures or segmentation-based measurements. Expert-defined IDPs and SSL-derived phenotypes have complementary strengths: IDPs provide direct clinical interpretability, reproducibility across processing pipelines, and established biological meaning, while SSL approaches capture broader and more distributed variation beyond predefined measurements and scale more readily to new imaging modalities, although interpretability and biological grounding remain challenges (Table 1). However, both approaches depend on image acquisition, preprocessing, quality control, technical choices, and cohort characteristics.

**Table 1:** Expert-Defined IDPs vs. SSL-Derived Phenotypes

Feature	Expert-Defined IDPs	SSL-Derived Phenotypes
Definition	Based on predefined anatomical segmentations and clinical measurements (e.g., hippocampal volume, LV ejection fraction, retinal layer thickness).	Learned from images using data-derived objectives; anatomical assumptions may still enter through preprocessing, registration, organ or region-of-interest selection, augmentations, and model architecture.
Susceptibility to technical and non-biological variation	May be affected by scanner, acquisition, segmentation, and processing differences, although targeted quality control can make these effects easier to identify.	May encode scanner or batch effects, demographic structure, preprocessing artifacts, and other nuisance variations, which can be difficult to distinguish from biological signals.
Interpretability	Generally directly interpretable; each phenotype maps to a known anatomical structure or clinical measure.	Abstract; biological meaning must be inferred post hoc through approaches such as saliency maps and perturbation.
Scalability to new modalities	Requires modality-specific segmentation pipelines and domain expertise for each new imaging type.	Can be applied to new modalities with minimal manual annotation.
Phenotypic coverage	Constrained by current anatomical knowledge; captures predefined features.	Can capture distributed, subtle morphological patterns beyond predefined measurements.
Multiple-testing burden	Usually involves fewer, predefined traits, resulting in a smaller multiple-testing burden, although correlated measurements still require correction.	Often produces many correlated latent dimensions, which increases the multiple-testing burden and motivates the use of dimensionality reduction or multivariate association methods.
Reproducibility	Standardized pipelines can support cross-study comparison, but reproducibility may vary across scanners, acquisition protocols, segmentation methods, software versions, quality-control procedures, and cohorts.	Dependent on training data, architecture, preprocessing, optimization, and cohort characteristics; cross-study reproducibility remains incompletely established.
Processing speed	Variable; minutes to hours per scan or subject for complex segmentation pipelines.	Fast at inference; seconds per image once trained.
Best suited for	Hypothesis-driven studies targeting known anatomical structures; clinical translation with established biomarkers.	Hypothesis-free discovery; identifying novel genetic associations beyond predefined anatomy; scaling to underexplored modalities.
Representative examples	Brain: hippocampal volume, cortical thickness, ventricular volume^1,29^; cardiac: left ventricular ejection fraction, left ventricular mass^2,81,82^; retina: retinal layer thickness, retinal vessel caliber^30,31^.	Brain: UDIPs from brain MRI^37^; cardiac: graph-autoencoder-derived cardiac shape phenotypes^59^; retina: iGWAS fundus embeddings^36^.

Although SSL is generally considered a form of unsupervised representation learning, it uses explicit predictive objectives whose targets are generated from the input data or naturally paired views, rather than from externally provided phenotype or trait labels. This distinguishes SSL from conventional unsupervised approaches such as clustering, dimensionality reduction, and density estimation. Although autoencoders were invented before the term SSL got popular, they are often categorized as an SSL method because the idea of deriving a compact representation (bottleneck) and using the decoder as a guide to train the encoder are very much in line with the philosophy of SSL. Our scope is defined by two inclusion criteria: (i) the method applies SSL directly to raw or minimally preprocessed medical images, rather than to pre-extracted expert-defined IDPs, and (ii) the learned representations are used as quantitative phenotypes for genome-wide association testing. This review aims to provide a methodological and biological framework for understanding how SSL-derived imaging phenotypes support genetic discovery. We searched PubMed and Google Scholar through June 2026 and screened the reference lists of included studies. Representative terms were combined: (“self-supervised learning” OR “unsupervised representation learning” OR autoencoder OR “contrastive learning”) AND (“medical imaging” OR MRI OR cardiac imaging OR retinal imaging OR fundus OR OCT) AND (“genome-wide association study” OR GWAS OR “genetic association”). We included studies that applied SSL or unsupervised representation learning directly to medical images and used the learned features as phenotypes for genome-wide association testing. We excluded studies that were based only on expert-defined IDPs, used supervised models without an SSL component, or did not include GWAS.

The review is organized as follows: we first summarize methodological foundations such as data preprocessing, SSL objectives and architectures, and strategies for converting embeddings into phenotypes and assessing their heritability and suitability for GWAS. We then review organ‑specific applications in brain MRI, cardiac MRI, and retinal imaging (fundus photography and optical coherence tomography (OCT)). Finally, we provide a forward-looking roadmap and discuss future directions. While challenges remain in reproducibility, cross-cohort generalization, interpretability, and biological validation, SSL-derived phenotypes represent a promising complement to expert-defined IDPs and may scale to biobank-level analyses; however, their broader adoption will require standardized benchmarking, external replication, and validation across diverse biobank and clinical cohorts.

## Overall framework of self-supervised learning approaches for genetic discovery

### SSL-based imaging genetics framework

**Fig. 1:**
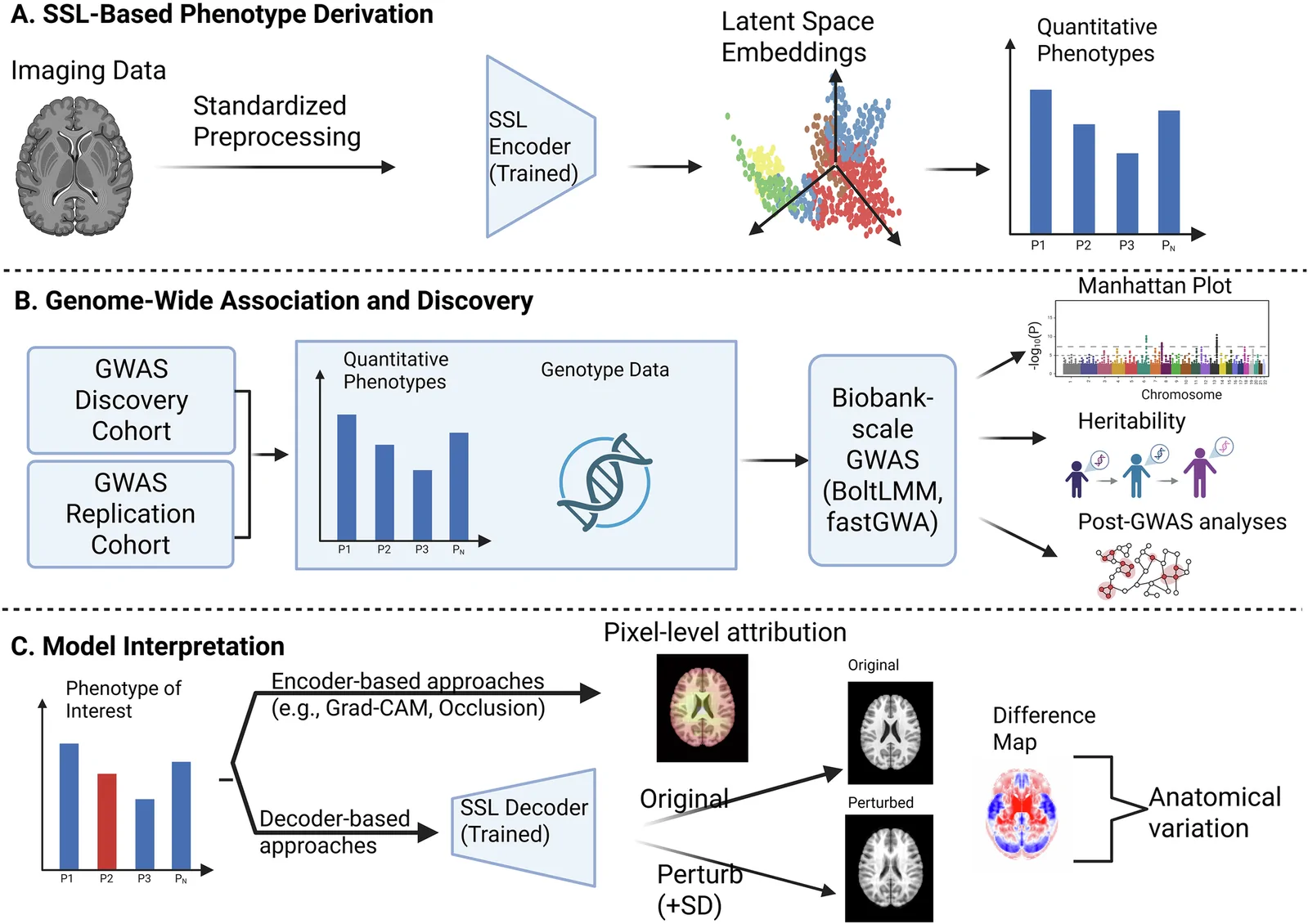
Overview of the self-supervised learning (SSL) imaging-genetics framework. **A** SSL-Based Phenotype Derivation: Medical images undergo standardized medical-image preprocessing steps. Input images are processed by a trained SSL encoder to extract high-dimensional latent space embeddings, which are harvested as quantitative phenotypes. **B** Genome-Wide Association and Discovery: Derived phenotypes are integrated with genotype data to perform GWAS. The phase yields summary statistics for Manhattan plots, SNP-based heritability estimates, and post-GWAS biological pathway analyses. **C** Model Interpretation: Strategies for mapping abstract latent phenotypes back to interpretable anatomical structures using either encoder-based or decoder-based approaches. UK Biobank images in this figure are reproduced with kind permission of UK Biobank ©. Brain MRI template image is derived from the MNI152/ICBM152 T1-weighted average structural MRI template.

To enable genetic discovery at biobank scale, we describe the SSL imaging genetics framework in three interconnected phases (Fig. 1): phenotype derivation, genome-wide association, and model interpretation.

#### Phase A: SSL-based phenotype derivation

The foundation of this framework, illustrated in Fig. 1A, is the derivation of robust imaging traits. Four datasets support an SSL imaging-genetics project: training and validation sets for model development and discovery and replication sets (with paired imaging and genetic data) for GWAS. Training sets in reviewed studies range from ~2500^40^ to ~32,000^36^, with most studies using several thousand images. A single biobank can be split into four non-overlapping subsets to control label leakage, or separate sources can be used for model development and GWAS^37^.

Before training SSL models, standardized preprocessing is necessary. For example, brain MRI typically requires skull stripping, registration to standard templates, bias-field correction, and intensity normalization^17^. Dynamic modalities such as cardiac MRI require additional temporal alignment across cardiac cycles^44^. The registration to a common template is especially important as it allows the model to ignore common features of all images and focus on individual variability instead.

For SSL imaging-genetics, a representation of the input image is harvested as the phenotype of interest. These phenotypes could be called endophenotypes: they are often heritable and thus are informative of the underlying biology behind the images. Autoencoders, neural networks designed to learn efficient data coding by training an encoder to compress the input and a decoder to reconstruct it, are frequently used^37^. Usually, the bottleneck layer between the encoder and the decoder is selected as the embedding of the input. This bottleneck represents the “latent space,” a compressed, multi-dimensional abstract representation of the original image. If the dimensionality (the number of variables in the latent space) is too large, an efficient set of phenotypes can be derived through dimensionality reduction methods such as principal component analysis^45,46^. Other approaches, such as R-indices, learn a small set of indices, with each index capturing a distinct pattern of anatomical variation rather than maximizing statistical variance, as PCA does^47^.

The resulting components of the latent space serve as the quantitative phenotypes (shown as P_1_…P_N_ in Fig. 1A) used for downstream analysis. These SSL‑derived phenotypes are often characterized via descriptive analyses to verify their meaningfulness. This validation typically includes visualization using approaches such as UMAP^48^ correlations with demographics (e.g., age, sex, BMI) and traditional IDPs using correlations and predictions and multivariate CCA analyses.

#### Phase B: Genome-wide association studies of SSL phenotypes

In the second phase, the derived latent representations are utilized as quantitative imaging phenotypes for genetic discovery. While GWAS methodology is well-established, SSL-derived phenotypes introduce specific analytical considerations. Unlike traditional IDPs, which are typically low-dimensional and clinically defined, SSL latent spaces can comprise tens to hundreds of correlated dimensions, creating challenges for multiple-testing correction and statistical power. The SSL-derived phenotypes are analyzed with standard linear or linear mixed‑model GWAS tools (e.g., BOLT‑LMM, fastGWA)^49,50^ (Fig. 1B). Given the high dimensionality and correlation among SSL features, multivariate tests (e.g., Multivariate Analysis of Variance (MANOVA)^51^) or joint/multi‑phenotype frameworks (e.g., Multivariate Omnibus Statistical Test (MOSTest)^52^ or Joint Analysis of multi-phenotype GWAS (JAGWAS)^53^) can aggregate modest effects across many traits and improve power. Post‑GWAS analyses are critical for interpreting and contextualizing association findings. Standard visualizations like Manhattan plots provide a genome-wide overview of significant loci. SNP‑based heritability estimates^37^ quantify the aggregate contribution of common genetic variants to phenotypic variance, indicating the extent to which the SSL-derived traits are genetically determined. Further translational steps, such as Mendelian randomization, help move beyond statistical association to infer potential causal relationships between imaging features and disease^54^.

#### Phase C: Model interpretation

Finally, interpreting SSL-derived features is essential because, unlike expert-defined IDPs that have built-in anatomical meaning (e.g., hippocampal volume, left ventricular mass), latent dimensions are abstract and their anatomical relevance must be inferred post hoc. For imaging models, linking phenotypes to localized image regions corresponding to specific anatomical landmarks is convenient to pinpoint the biological structures corresponding to a latent dimension (Fig. 1C). Encoder-based approaches, such as saliency maps (e.g., Grad-CAM and Integrated Gradients), help achieve this by highlighting pixel-level regions that influence specific latent dimensions^55–58^. Furthermore, decoder-based perturbation approaches provide a direct visualization by decoding latent vectors perturbed along dimensions associated with significant loci; researchers can observe the precise anatomical variations driven by specific genetic variants^37,59^. Conversely, for multivariate GWAS frameworks (e.g., MOSTest) that aggregate signals across multiple phenotypes reflecting distributed anatomical variations, mapping individual dimensions is less relevant^52^. For instance, MOSTest applied to regional brain morphology has revealed variants driving distributed gradients rather than localized effects. Instead, regression networks trained on embeddings can predict known traits (e.g., sulcal depth); averaging anatomical data from individuals at the prediction extremes visualizes the consensus structural variation driving the multivariate association^60^. Alternatively, association-based difference maps can localize morphological features by linking latent dimensions to specific conditions (e.g., disease status) and visualizing the pixel-wise differences between the average images of cases and controls^40^.

Unlike most traditional medical imaging AI applications that are typically dealing with modest sample sizes, imaging GWAS requires large sample sizes to achieve sufficient statistical power. As a result, efficient computational strategies are required for scalable data processing, model development, and evaluation. Beyond traditional pipelines, deep learning frameworks offering GPU-accelerated model training and efficient data handling, such as NVIDIA Data Loading Library (DALI), are becoming available^61–63^.

## Evaluating the added value of SSL-derived phenotypes beyond conventional IDPs

SSL-derived phenotypes are most likely to provide information beyond conventional IDPs when relevant imaging variation is distributed across an organ or involves complex shape, texture, spatial relationships, or temporal dynamics that cannot be summarized adequately by a small set of predefined measurements. However, added value should not be inferred solely from the number of genome-wide significant loci, because locus counts may also reflect differences in phenotype dimensionality, sample size, statistical power, or definitions of novelty. Ideally, SSL-derived phenotypes and conventional IDPs should be compared in the same participants under similar analytical conditions, with complementarity shown by reproducible genetic associations that persist after accounting for established IDPs. Biological or clinical interpretation requires additional evidence such as feature stability, independent genetic replication, functional support, or associations with meaningful clinical traits and outcomes.

The following section describes the principal SSL approaches through which these complementary imaging features may be derived.

### Self-supervised learning approaches

**Fig. 2:**
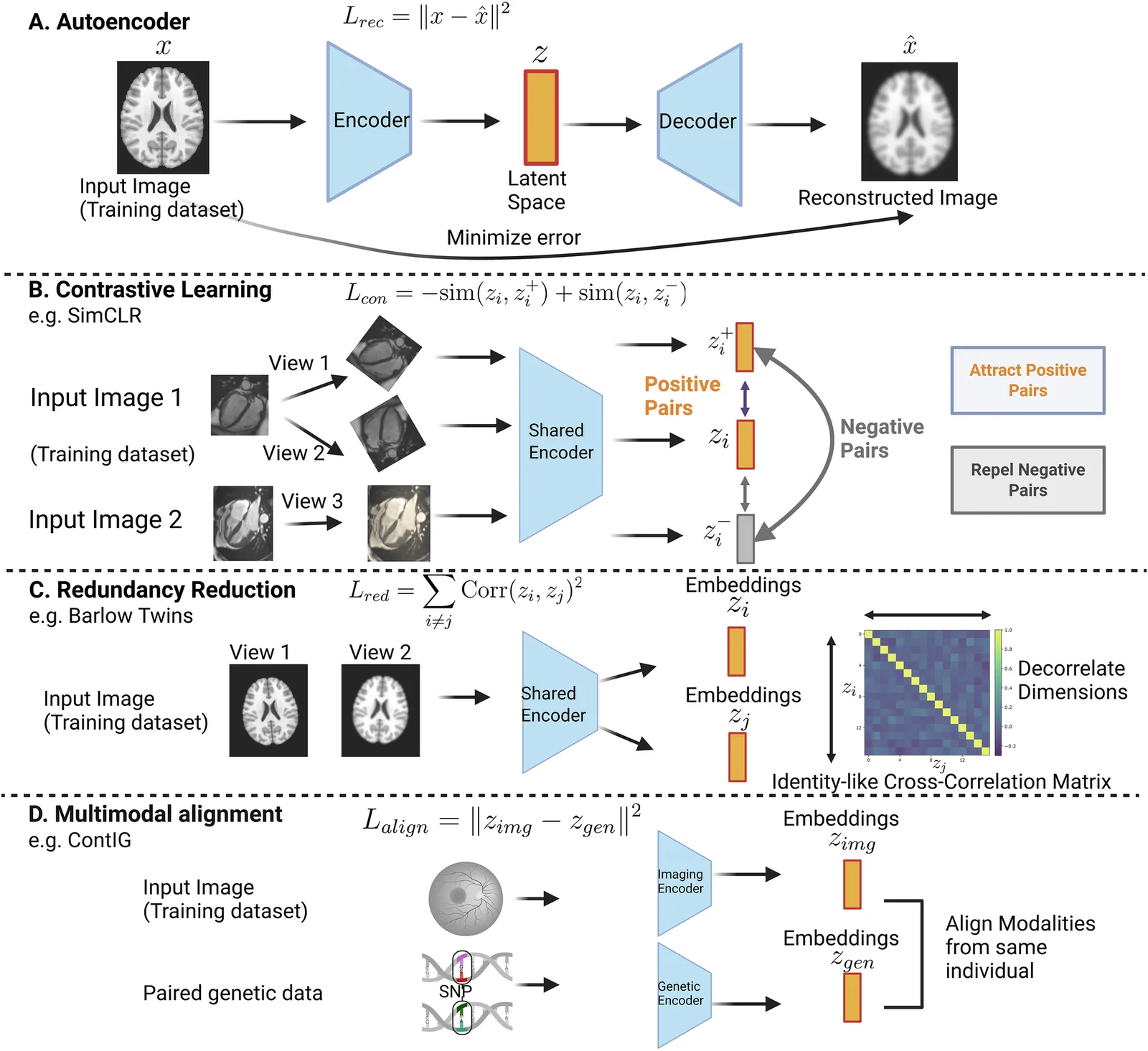
Overview of self-supervised learning families used for imaging-genetics phenotyping. **A** Autoencoder (reconstruction-based). An encoder compresses the input image *x* into a low-dimensional latent *z*, and a decoder reconstructs it as *x̂*; training minimizes reconstruction error *L*_rec_ = ‖*x* − *x̂*‖^2^. **B** Contrastive learning (e.g., SimCLR). Two augmented views of the same image (positive pair) are mapped through a shared encoder and pulled together in latent space, while views from different images (negative pair) are pushed apart, with loss *L*_con_ = −sim(*z*_i_, *z*_i_^+^) + sim(*z*_i_, *z*_i_^−^). **C** Redundancy reduction (e.g., Barlow Twins). Two augmented views are encoded, and the cross-correlation matrix between their embedding dimensions is driven toward identity, decorrelating dimensions while keeping them invariant across views: *L*_red_ = Σ_i≠j_ Corr(*z*_i_, *z*_j_)^2^. **D** Multimodal alignment (e.g., ContIG). Modality-specific encoders embed paired imaging and genetic data from the same individual into a shared latent space, with alignment enforced by minimizing *L*_align_ = ‖*z*_img_ − *z*_gen_‖^2^. UK Biobank images in this figure are reproduced with kind permission of UK Biobank ©. Brain MRI template image is derived from the MNI152/ICBM152 T1-weighted average structural MRI template.

For model development, we categorize SSL strategies in imaging genetics into two primary families: reconstruction-based and joint-embedding approaches (Fig. 2; Table 2). Throughout this Review, SSL encompasses reconstruction-based and joint-embedding methods in which the supervisory signal is derived from the input data itself or from naturally paired views or modalities; this includes methods historically described as unsupervised, such as autoencoders, and hybrid studies when they contain an SSL component. Despite their architectural differences, both share a common goal: learning informative, low-dimensional representations while avoiding representational collapse, the failure mode in which the encoder maps all inputs to a trivial, constant output. Each family addresses this challenge through a different mechanism.

**Table 2:** GWAS-relevant comparison of major self-supervised learning approaches in imaging genetics

Feature	Reconstruction-based (AE, VAE, MAE)	Joint embedding: instance discrimination Same image, two views (SimCLR, MoCo, Barlow Twins, VICReg)	Joint embedding: cross-modal alignment Same individual, two modalities (ContIG, Genetic InfoMax, CMAE)
What representation is encouraged?	Retain enough information to reconstruct the input through a low-dimensional bottleneck.	Embeddings of related views (typically augmented views of the same image) should agree while preserving the variability that distinguishes one individual from another.	Embeddings from paired modalities of the same individual (e.g., image + genetics) should align in a shared latent space.
Formal training objective	*L*_rec_ = ‖x − x̂‖² VAE adds a Kullback–Leibler regularizer that pulls the latent toward a Gaussian prior. MAE restricts the loss to randomly masked image patches.	**Contrastive** (Fig. 2B)**:** L_con_ = −sim(z_i_, z_i_⁺) + sim(z_i_, z_i_⁻) **Decorrelation/Barlow Twins** (Fig. 2C)**:** *L*_red_ = Σ_i≠j_ Corr(z_i_, z_j_)² VICReg replaces the correlation term with separate variance, invariance, and covariance terms.	*L*_align_ = ‖z_img_ − z_gen_‖² Or a contrastive variant in which negative pairs are formed across individuals.
Conceptual interpretation	The model is penalized whenever the reconstructed image differs from the original, so the encoder is forced to pack the most informative anatomical content into the bottleneck.	The loss shrinks when two views of the same image (or same individual) sit close in latent space and far from views of others, while latent dimensions are encouraged to carry independent, non-redundant information.	The loss shrinks when imaging and genetic embeddings of the same person sit close together in a shared latent space, so the two encoders are trained to agree on a common per-person representation.
How collapse is avoided	A trivial constant output cannot reconstruct the input; the reconstruction requirement itself prevents collapse.	By repulsion from negative pairs (SimCLR, MoCo), by an explicit decorrelation term across latent dimensions (Barlow Twins, VICReg), or by architectural asymmetry with a momentum target encoder (BYOL).	By the requirement to preserve identity-level distinguishing information; collapsing all individuals to the same embedding would defeat the cross-modal matching task.
Examples in reviewed studies	UDIPs: 3D autoencoder on brain MRI^37^; Autoencoder-OCT: autoencoder on retinal thickness maps^40^; TransferGWAS: VAE trained on ADNI brain scans^46^; Graph-convolutional autoencoder ensemble on LV meshes^59^	iGWAS: contrastive learning on left–right retinal fundus pairs^36^; Champollion V0: Barlow Twins on ACC sulcal patterns^60^; SimCLR baselines used for comparison in ContIG^55^.	ContIG: aligning retinal fundus images with genetic vectors^55^; Genetic InfoMax: aligning brain MRI with genetic data^45^; Cross-Modal Autoencoder: aligning ECG with cardiac MRI^74^.
GWAS-relevant strengths	Captures rich anatomical variation through the bottleneck. The decoder enables interpretation by perturbation-based decoding (latent dimensions can be visualized as image changes). Broadly suited for hypothesis-free discovery across organs.	Decorrelation-based variants (Barlow Twins, VICReg) yield latent dimensions that are uncorrelated by design, with potential affinity for multivariate GWAS frameworks (e.g., MOSTest), where decorrelated phenotypes can reduce the effective number of statistical tests. Contrastive variants can suppress nuisance variation that is consistent across augmentations.	For genotype-informed methods such as ContIG and Genetic InfoMax, genetic information is incorporated during representation learning, which may enrich genetically relevant variation. Joint use of two modalities can recover variants missed by single-modality analyses.
GWAS-relevant trade-offs	Latent dimensions are typically correlated; the bottleneck may retain unwanted variation such as scanner or acquisition artifacts; high-dimensional latents inflate the multiple-testing burden.	Augmentation invariance may erase heritable signal if the chosen augmentations destroy it. Decorrelation-based variants lack a built-in decoder for anatomical attribution. Empirical comparisons against reconstruction baselines on identical data are scarce.	For genotype-informed methods such as ContIG and Genetic InfoMax, using genetics in the training objective creates potential circularity if the same individuals (or related pairs) are included in both model training and subsequent GWAS. Non-overlapping representation-learning and GWAS samples are therefore needed to avoid inflated or difficult-to-interpret association and heritability estimates. Cross-modal approaches also require paired data from the same individuals.

SSL families are organized as reconstruction-based versus the joint-embedding family. Within joint embedding, two practical sub-variants are distinguished: instance discrimination (where the matched pair is two augmented views of the same image) and cross-modal alignment (where the matched pair is two modalities of the same individual). For each, the table lists the learning objective, a plain-language reading of the loss anchored to Fig. 2, the collapse prevention mechanism, representative studies in the review, and GWAS-relevant strengths and trade-offs.

Notation. x = input image; x̂ = reconstructed input; z = learned embedding (latent vector); zᵢ, zⱼ = embeddings of paired views/modalities; zimg, zgen = imaging and genetic embeddings of the same individual; sim(·,·) = cosine similarity between two embeddings; Corr(·,·) = correlation across the batch between matched dimensions of the two views; ‖·‖² = squared Euclidean distance.

*ACC* anterior cingulate cortex, *ADNI* Alzheimer’s Disease Neuroimaging Initiative, *AE* autoencoder, *BYOL* Bootstrap Your Own Latent, *CMAE* Cross-Modal Autoencoder, *ContIG* Contrastive Imaging and Genetics, *ECG* electrocardiogram, *GWAS* genome-wide association study, *LV* left ventricle, *MAE* masked autoencoder, *MoCo* Momentum Contrast, *MOSTest* Multivariate Omnibus Statistical Test, *MRI* magnetic resonance imaging, *OCT* optical coherence tomography, *SimCLR* Simple Framework for Contrastive Learning of Visual Representations, *SSL* self-supervised learning, *UDIPs* Unsupervised Deep-Learning-Derived Imaging Phenotypes, *VAE* variational autoencoder, *VICReg* Variance-Invariance-Covariance Regularization.

Reconstruction-based methods learn representations by compressing an input through a low-dimensional bottleneck and then reconstructing it (Fig. 2A). To succeed under this constraint, the model must filter out noise and redundancy and retain the most salient structural and semantic information. The resulting bottleneck representation, not the reconstruction itself, is what is used as a GWAS phenotype. A classic autoencoder maps an input x to a latent vector *z* = f_θ_(x) and decodes it back to x̂ = g_ϕ_(z), training to minimize reconstruction error: *L*_rec_ = ‖ x − x̂ ‖^2^^2^. In plain terms, the model is penalized whenever the reconstructed image differs from the original (Fig. 2A), so the encoder is forced to pack the most informative anatomical content into the latent bottleneck^37,64^.

Autoencoders trained this way have been shown to be effective phenotypers for brain images. Variational autoencoders (VAEs) extend this objective by adding a Kullback-Leibler term that regularizes the latent distribution toward a Gaussian prior, encouraging smoother latent geometry^39,65^. Masked autoencoders (MAEs) instead reconstruct only randomly masked patches of the input, forcing the network to learn rich semantic context and anatomical shape priors to predict the missing tissue^66^.

Joint-embedding methods take a different approach: rather than reconstructing the input, they learn a logical relationship among embeddings produced by parallel encoders applied to different views or modalities of the same underlying subject (Fig. 2B–D). Given encoders *f*_θ_ and *f*_θ′_, paired inputs (*x*_i_, *x*_j_) are mapped to embeddings *z*_i_ = *f*_θ_(*x*_i_) and *z*_j_ = *f*_θ′_(*x*_j_). The choice of loss determines what relationship the embeddings should encode and divides this family into three branches: contrastive (Fig. 2B), redundancy-reduction (Fig. 2C), and multimodal alignment (Fig. 2D).

Contrastive learning captures features shared across variations of the same input by maximizing similarity between positive pairs, typically two augmented views of the same image, or naturally paired data such as left-right retina, while pushing apart negative pairs drawn from different individuals^36,67,68^ (Fig. 2B). At its simplest, the loss attracts an anchor toward its positive partner and repels it from a negative: *L*_con_ = −sim(*z*_i_, *z*_i_^+^) + sim(*z*_i_, *z*_i_^−^). In plain terms, this loss shrinks when the anchor embedding sits close to its positive partner and far from negative examples, thereby pulling together views of the same individual while pushing apart views from different individuals (Fig. 2B).

SimCLR formalizes this idea using the InfoNCE objective, which contrasts each anchor against all negatives in the batch via a softmax over similarities; this approach requires very large batches to supply enough negative pairs for stable training^68^. MoCo relaxes this requirement by maintaining a memory bank of past representations as negatives, decoupling negative-sample count from batch size^69^.

Redundancy-reduction methods avoid negative samples entirely. Barlow Twins pass two augmented views of the same image through a shared encoder and decorrelate their embeddings so that each latent dimension captures unique, non-redundant information (Fig. 2C)^60,70^. The objective minimizes the squared off-diagonal correlations between embedding dimensions of the two views: *L*_red_ = Σ_i≠j_ Corr(*z*_i_, *z*_j_)^2^^2^. In plain terms, the loss is small when different latent dimensions carry independent, non-redundant information, driving the cross-correlation matrix between the two views toward an identity-like form (Fig. 2C).

This simultaneously enforces invariance across views and decorrelation across latent dimensions. Related approaches share this redundancy-reduction philosophy: VICReg combines variance, invariance, and covariance terms to prevent collapse^71^. BYOL avoids collapse using an asymmetric architecture with a momentum-updated target encoder and a predictor head rather than an explicit decorrelation term^72^.

Multimodal alignment extends joint embedding across distinct input streams (Fig. 2D). CLIP (Contrastive Language-Image Pre-Training) established the foundational framework by training paired image and text encoders with a contrastive objective^73^, and the same paradigm has since been adapted to imaging-genetics, for example, aligning retina images with genetics or electrocardiogram (ECG) with cardiac MRI into a shared latent space^55,74^. Genetic-guided approaches such as ContIG directly align an individual’s image and genetic embeddings^55^, in which case a separate GWAS data set is required to avoid label leakage. We classify genotype-informed methods such as ContIG and Genetic InfoMax as multimodal SSL because matched imaging and genetic data from the same individual serve as the supervisory signal without requiring manually annotated phenotype labels. When genotype data contribute to representation learning, using the same individuals for model training and subsequent GWAS can create circularity and information leakage because the tested genotypes have already influenced the phenotype definition. Representation-learning and GWAS cohorts should therefore be non-overlapping, and any sample overlap should be reported explicitly. The simplest alignment objective minimizes squared distance between paired embeddings: *L*_align_ = ‖ *z*_img_ − *z*_gen_‖^2^^2^. In plain terms, the loss is small when imaging and genetic embeddings of the same individual sit close together in the shared latent space, so the two modality-specific encoders are jointly trained to agree on a common representation per person (Fig. 2D). In practice, contrastive or auxiliary losses are often needed to preserve individual-level variation and avoid representational collapse.

Reconstruction-based and joint-embedding approaches are not mutually exclusive, and several studies in this review combine them. Genetic InfoMax pairs image reconstruction with contrastive alignment between MRI and genetic embeddings^45^, while the cross-modal autoencoder combines modality-specific reconstruction losses with a contrastive loss across ECG and cMRI^74^. These objectives differ in trade-offs relevant to GWAS (Table 2), and resolving which combinations work best will require head-to-head benchmarks on shared datasets with standardized pipelines. To highlight the main conceptual and GWAS-relevant differences across these SSL families, we provide a cross-method comparison in Table 2.

**Fig. 3:**
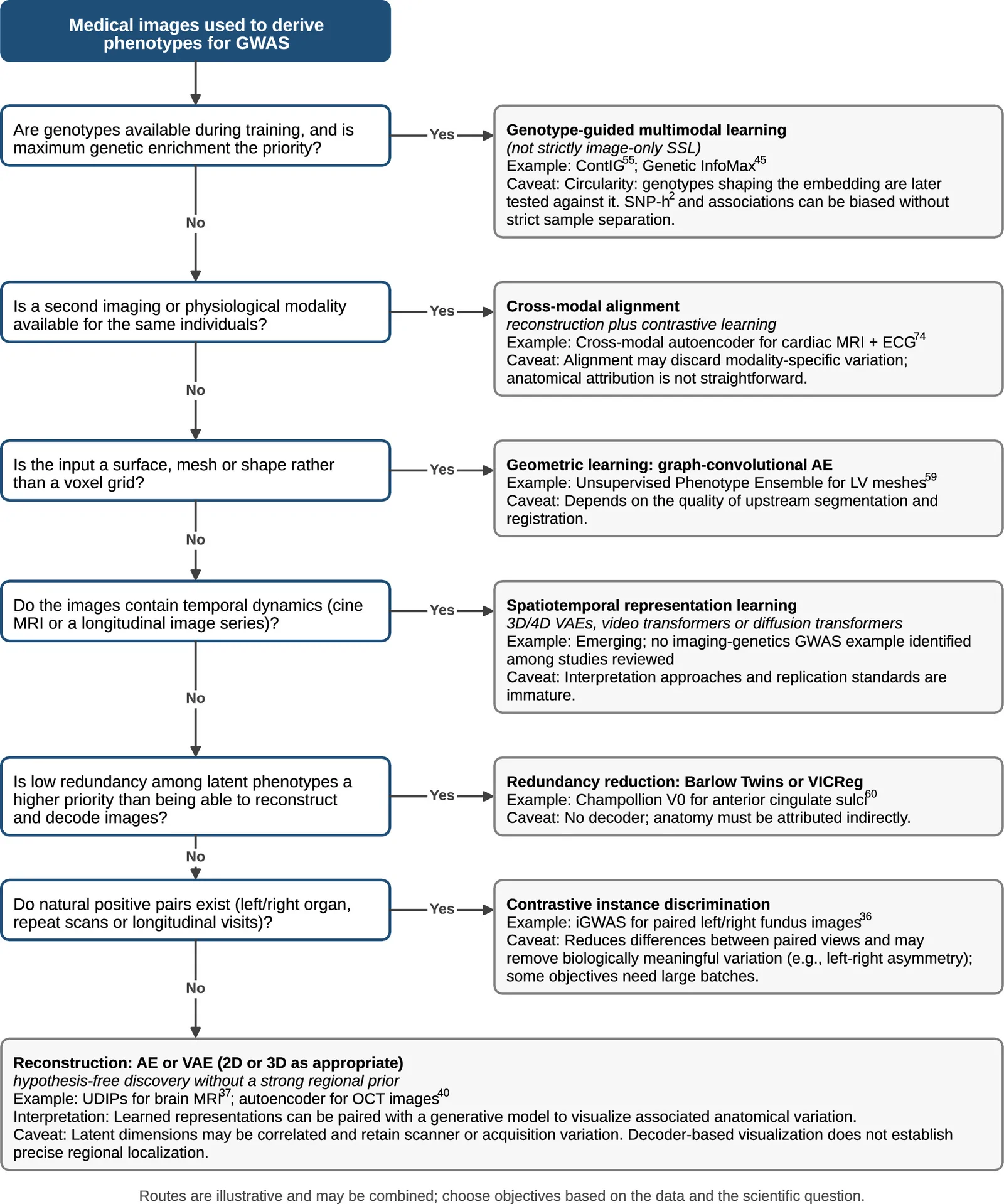
Decision map for selecting self-supervised learning approaches for imaging-phenotype GWAS. Each question is answered in turn; a “Yes” leads to the recommended approach (gray box) with a representative implementation and its principal limitation, and a “No” proceeds to the next question. When no question applies, the default is reconstruction-based autoencoding. Superscript numbers refer to the reference list. AE autoencoder, ECG electrocardiogram, GWAS genome-wide association study, LV left ventricle, MRI magnetic resonance imaging, OCT optical coherence tomography, SNP-h² SNP-based heritability, SSL self-supervised learning, UDIPs Unsupervised Deep-Learning-Derived Imaging Phenotypes, VAE variational autoencoder, VICReg Variance-Invariance-Covariance Regularization.

The choice of SSL approach should be guided by the biological question, imaging modality, available sample size, data structure, and desired level of phenotype interpretability. Fig. 3 provides a practical decision map linking common study settings to suitable SSL strategies and summarizes their principal advantages, limitations, and GWAS-specific considerations.

This decision map is intended as practical guidance rather than a strict classification, as the optimal approach will also depend on cohort characteristics, computational resources, and validation data.

We next review organ‑specific applications of SSL in imaging genetics. For each organ, we summarize datasets and preprocessing, the SSL objectives and architectures used, how embeddings were converted to phenotypes and validated (including SNP‑heritability), key genetic findings and replication, interpretability analyses, and limitations.

## Brain imaging genetics

Traditional brain imaging-genetics studies have relied on expert-defined IDPs, ranging from volumetric measures like hippocampal volume and cortical thickness using software packages like FreeSurfer and FSL^1,29^ to high-dimensional voxel-wise maps derived from Tensor-Based Morphometry (TBM) and Voxel-Based Morphometry (VBM)^75,76^. While successful, these approaches face inherent trade-offs: segmentation algorithms are sensitive to image quality and anatomical priors limited by existing anatomical knowledge, while voxel-wise methods suffer from severe multiple-testing burdens that reduce statistical power. SSL approaches address these limitations by learning compact, data-driven phenotypes directly from the images (Table 3).

**Table 3:** Summary of self-supervised learning approaches across brain, cardiac, and retinal imaging genetics

Study	Imaging modality	SSL approach	Architecture	Discovery/replication N (UKBB)	Loci (discovery/replication)	Novel loci^a^
**Brain imaging**						
**UDIPs**^37^	T1/T2 MRI	Reconstruction (autoencoder)	3D CNN	22k/12k	97/60	26
**UDIP-FA**^77^	Diffusion MRI FA maps	Reconstruction (autoencoder)	3D CNN	17.4k/8.5k	300/182 lead SNPs^b^	Not quantified
**GIM**^45^	T1 MRI	Multimodal alignment (image + genetics)	3D CNN + genetic transformer	28.5k/ –	40/ –	15
**TransferGWAS**^46^	T1 MRI	Transfer learning (ImageNet & ADNI) + reconstruction	3D CNN VAE (ADNI) + 2D CNN ResNet-50 (ImageNet)	36.3k/ –	289 (union)^c^/ –	11
**Champollion V0**^60^	T1 MRI	Redundancy reduction (Barlow Twins) on anterior cingulate sulcal region	3D CNN	36k/5.4k	14/1	Not quantified relative to atlas
**Cardiac imaging**						
**Cross-Modal Autoencoder (CMAE)**^74^	cMRI + 12-lead ECG	Cross-modal autoencoder (reconstruction + contrastive)	1D CNN (ECG) + 2D CNN (MRI)	38.7k/ –	93 joint, 86 ECG, 33 MRI (lead SNPs)^b^/ –	~70–90 SNPs per trait beyond univariate GWAS
**Unsupervised Phenotype Ensemble (UPE)**^59^	cMRI	Graph-convolutional autoencoders (ensemble) on 3D LV meshes	Graph CNN (spectral Chebyshev), 36-model ensemble	48.7k/5.5k	49/28	40
**Retinal imaging**						
**iGWAS**^36^	Fundus photographs	Contrastive learning (ArcFace, L/R eye pairs)	Inception V3 CNN	65.6k (130.3k images)/L-R consistency only	14/ –	3
**ContIG**^55^	Fundus photographs	Multimodal contrastive (image + genetics)	ResNet-50 CNN + MLP	7.1k/ –	16–22/ –	Not quantified
**TransferGWAS (Retina)**^86^	Fundus photographs	Transfer learning (ImageNet + EyePACS pretraining)	ResNet-50 CNN	46.7k/ –	60/ –	7
**Autoencoder-OCT**^40^	OCT thickness maps	Reconstruction (autoencoder)	2D CNN autoencoder	31.1k/10.4k	118/41	20

*ADNI* Alzheimer’s Disease Neuroimaging Initiative, *CMAE* Cross-Modal Autoencoder, *cMRI* cardiac magnetic resonance imaging, *CNN* convolutional neural network, *ECG* electrocardiogram, *FA* fractional anisotropy, *GIM* Genetic InfoMax, *GWAS* genome-wide association study, *IDP* image-derived phenotype, *L/R* left/right, *LV* left ventricle, *MLP* multi-layer perceptron, *MRI* magnetic resonance imaging, *OCT* optical coherence tomography, *SNP* single nucleotide polymorphism, *SSL* self-supervised learning, *UDIP* Unsupervised Deep-learning Derived Imaging Phenotype, *UKBB* UK Biobank, *UPE* Unsupervised Phenotype Ensemble, *VAE* variational autoencoder.

^a^Novel-locus definitions vary by study: UDIPs = loci not found in prior UKBB IDP GWAS; GIM = loci unique to GIM relative to other SSL baselines tested in the study; TransferGWAS (brain) = loci with no previously reported associations in the NHGRI-EBI GWAS Catalog; Champollion V0 did not quantify novelty against an atlas reference; CMAE = SNPs detected by the joint multivariate model but missed by univariate GWAS of single ECG traits (PQ, QRS, QT, QTc, RR); UPE = loci not previously reported in standard LV GWAS; iGWAS = loci with no prior associations with eye measures, eye diseases, or pigmentation in the GWAS Catalog; ContIG = novelty not explicitly quantified; TransferGWAS (retina) = loci with no previously reported associations with eye-related, pigmentation, or major systemic traits in the GWAS Catalog; Autoencoder-OCT = loci not previously reported in large-scale GWAS of traditional expert-defined OCT phenotypes.

^b^Lead SNPs rather than independent loci. UDIP-FA identified 420 lead SNPs across 300 loci in the discovery cohort but assessed replication only at the lead-SNP level (182 of 420 lead SNPs replicated). CMAE reported lead SNPs only and did not perform locus-level clumping.

^c^TransferGWAS used the full sample (*N* = 36.3k) for discovery to maximize power and did not report a held-out replication. A separate split (discovery *N* = 23.6k; replication *N* = 12.7k) replicated 70 loci.

Early applications focused on reconstruction-based methods. Unsupervised Deep-Learning Derived Imaging Phenotypes (UDIPs) employed three-dimensional convolutional autoencoders to encode T1-weighted or T2-FLAIR scans into 128-dimensional vectors optimized purely for image reconstruction^37^. Trained on 6130 mixed-ethnicity UKBB participants, the model derived latent phenotypes for GWAS in a larger discovery (*n* ~ 22,000) and replication (*n* ~ 12,000) cohort. The analysis identified 97 independent loci, 60 of which replicated, including 26 novel loci not captured by traditional IDP GWAS^1^. A subsequent meta-analysis of the discovery and replication cohorts expanded the findings to 199 significant loci; notably, 145 of these were not found in traditional IDP GWAS, including 29 novel loci previously unrelated to any brain-related traits. The average SNP-heritability of UDIPs (*h*² ≈ 0.25) exceeded that of expert-defined traits (*h*² ≈ 0.18). By decoding perturbed latent vectors back into image space, the authors visualized the specific anatomical structures (e.g., ventricles, thalamus) driving genetic associations. However, perturbation of a single latent dimension can propagate globally in the decoded image, making anatomical attribution less precise than voxel-wise IDPs.

A related extension applied the UDIP framework to diffusion MRI fractional anisotropy (FA) maps to capture white matter microstructure^77^. Using a 3D convolutional autoencoder trained on voxel-wise FA maps, the study derived 128-dimensional UDIP-FA embeddings and used them as quantitative phenotypes. Multivariate GWAS identified 420 lead SNPs across 300 loci in the discovery cohort, of which 182 lead SNPs replicated; meta-analysis of both cohorts identified 939 lead SNPs across 586 loci. Using a perturbation-based decoder approach similar to the original UDIP study, the authors perturbed individual UDIP-FA dimensions and decoded them back into image space to generate voxel-wise t-maps, which were then analyzed for enrichment across ICBM DTI-81 white-matter tracts. These findings suggest that SSL-derived phenotypes can extend beyond structural T1/T2 MRI to microstructural diffusion MRI.

In contrast, Genetic InfoMax utilized a multimodal contrastive learning framework to align image and genotype data^45^, in addition to an image reconstruction task. Using a 3D CNN encoder for MRI and a novel transformer-based genetic encoder for SNPs, GIM was trained to maximize mutual information (InfoMax) between the MRI and genetic embeddings. Genetic InfoMax reported a heritability of *h*² ≈ 0.38 and improved genetic discovery with 40 loci (15 unique to this method) for the top 10 principal components (PCs) of the endophenotypes. However, unlike UDIPs, GIM did not include a dedicated interpretability pipeline or replication cohort, and the choice of using top PCs as a phenotype was meant for methodological development rather than maximizing biological discovery. Therefore, further rigorous studies at scale are warranted.

Another example is TransferGWAS^46^ which derived phenotypes using two distinct strategies: a 3D CNN VAE trained on ADNI scans with a self-supervised image-reconstruction objective combined with supervised dementia prediction and a 2D ResNet-50 pretrained on ImageNet natural images via supervised transfer learning^78^. The study identified a union of 289 loci (165 from ADNI-derived phenotypes and 194 from ImageNet-derived phenotypes, with a shared overlap of 70 loci). The ADNI-derived phenotypes captured brain-specific variation (mean h² = 0.19), and ImageNet traits reflected systemic features (mean *h*² ≈ 0.13). This shows SSL phenotypes may capture systemic whole-body traits (e.g., bone density, BMI) rather than brain-specific mechanisms, necessitating rigorous downstream validation. The higher number of loci reported by Rakowski et al.^46^ is primarily driven by their significantly larger discovery sample size (*N* = 36,311 vs. UDIPs *N *= 22,800) and reduced multiple-testing burden (20 phenotypes vs. UDIP 256 phenotypes with T1/T2), combined with their capture of non-brain signals.

Dufournet et al. trained a Barlow‑Twins style CNN-based model (“Champollion V0”)^79^ to encode local sulcal morphology within the anterior cingulate cortex (ACC) from UKBB T1 MRI^60^. Using BrainVISA/Morphologist^80^ to extract sulcal skeletons and an ACC region of interest, the model learned a 256‑dimensional latent space on 36,000 White‑British participants. This cohort served as both the training and discovery set, while a distinct non‑White‑British cohort (*n* = 5395) was used for replication. Principal components explaining 99.9% of variance yielded 42 PCs (left ACC) and 39 PCs (right ACC) for association testing with the MOSTest multivariate GWAS framework. In discovery, the authors reported 10 loci (left ACC) and 4 loci (right ACC) linked to specific ACC folding patterns; one locus replicated nominally in the smaller replication cohort. For interpretation, they used regression-guided visualizations by training linear models to predict specific sulcal traits (e.g., presence of the paracingulate sulcus) from the full latent space. By identifying the subjects falling at the extremes of these prediction probabilities, they computed the average of the 3D sulcal skeletons for the high-probability group versus the low-probability group and then visualized the resulting consensus anatomical configurations (e.g., double-parallel patterns). This approach provides a direct visual link between the abstract latent features and the biological structures associated with the discovered genetic loci. Unlike UDIPs, which map individual latent dimensions to brain regions, this method interprets the multivariate direction of effect consistent with the MOSTest discovery framework.

Collectively, these approaches highlight key trade-offs in SSL-based imaging genetics: reconstruction-based models (e.g., UDIPs) offer broad anatomical coverage and a clear interpretation framework, whereas transmodal contrastive/InfoMax methods (e.g., GIM) can yield higher heritability. Transfer learning strategies (e.g., TransferGWAS) may reduce the amount of data required for model training, leaving more samples available for GWAS to increase discovery power, but domain shift across data sets could potentially reduce the strength of the phenotyping mode. Here, domain shift refers to differences in scanner hardware, acquisition protocols, preprocessing pipelines, and cohort characteristics across datasets. Region-focused models (e.g., Champollion) suggest that local cortical folding phenotypes may have distinct, but sometimes subtler, genetic architectures.

Overall, SSL brain imaging genetics studies are still few and far between. Existing methods all demonstrated the power of identifying new loci beyond traditional IDP-based GWAS. However, these methods rely on different preprocessing pipelines (e.g., affine vs. non-linear registration), different GWAS sample sizes, different numbers of phenotypes, and different loci clumping protocols (Table 3), making direct cross-study comparison of GWAS results difficult.

## Cardiac imaging genetics

Traditional cardiac imaging-genetics studies rely on traditional IDPs such as left ventricular (LV) mass, end-diastolic volume, left ventricular ejection fraction, and wall thickness measurements extracted using automated analysis pipelines^81,82^. While clinically validated, these IDPs may fail to capture the full complexity of cardiac morphology, including regional shape variations and subtle geometric deformations. Furthermore, although cardiac MRI (cMRI) provides rich 4D (3D spatial + 1D temporal) data, standard analyses typically reduce this to static measurements at end-diastole and end-systole. SSL approaches could potentially capture these comprehensive phenotypes, though current methods remain limited by their focus on static snapshots and training cohorts of largely European ancestry (Table 3).

An early application was a cross-modal autoencoder framework integrated cMRI with ECG data from 38,686 UKBB participants^74^. The approach used separate 1D and 2D CNN autoencoders for ECG and cMRI, respectively, with a shared 256-dimensional latent space. Training combined reconstruction losses for each modality with a contrastive loss that encouraged paired ECG-cMRI samples to have similar representations while separating unpaired samples. While cMRI data included 50 frames across the cardiac cycle, the 2D CNN architecture treats these temporal frames as separate channels rather than processing sequential temporal dynamics, limiting exploitation of true spatiotemporal cardiac motion patterns. Using MANOVA, the joint model identifies more lead SNPs (93) than the MRI-only model (33) and the ECG-only model (86), indicating it captures signals missed when using only one modality’s representation. For genetic discovery, a MANOVA analysis of the multivariate latent space identified variants affecting cardiovascular physiology that were missed by single-modality analyses. Regarding interpretation, the study did not map genetic associations to localized anatomical regions. However, the authors demonstrated that the learned representations captured relevant physiological information by predicting demographics (age, sex, BMI) and traditional phenotypes (e.g., Left Ventricular Mass [LVM] and Right Ventricular End-Diastolic Volume [RVEDV]) from single modalities^74^. Also, they showed it is possible to impute synthetic MRI video frames from ECG inputs, where metrics derived from the synthetic images correlated with ground truth values (e.g., LVM and RVEDV). The cross-modal integration represents a methodological advance, though the genetic discoveries largely involved known cardiovascular genes such as *NOS1AP*, *TTN*, *SCN10A, and SLC35F1*^74^.

A geometric deep learning approach applied graph convolutional autoencoders to LV shape analysis using 3D surface meshes from 48,651 UKBB participants (discovery) with replication in 5470 participants^59^. Preprocessing involved automatic segmentation to generate registered 3D LV surface meshes (~5k vertices) at end diastole. The method focused on the end-diastole phase only and used spectral Chebyshev convolutions^83^, a kind of graph convolutional network (GCN), to learn shape representations while preserving mesh topology. An ensemble approach pooled representations from 36 models trained with different hyperparameters, yielding 324 latent features after correlation-based filtering. GWAS identified 49 genome-wide significant loci, with 40 claimed as novel. Interpretability was achieved through visual shape effect analysis: for each significant genetic locus, they identified the most strongly associated latent variable and visualized heart meshes across quantile ranges [0–1%, 9.5–10.5%, 49.5–50.5%, 89.5–90.5%, 99–100%] to demonstrate how genetic variants affect specific 3D morphological patterns. For example, the *STRN* locus was associated with a change in mitral valve orientation without altering LV volume.

These studies demonstrate that SSL can uncover novel loci in cardiac imaging genetics, but they represent only sparse attempts at exploration. More comprehensive studies are needed with external replication, diverse cohorts, and systematic comparisons against standard cardiac IDPs to fully unlock the potential of SSL heart imaging genetics. Also, challenges in generalizability remain. Both pipelines were trained on 1.5 T Siemens scans from the UKBB^84^, leaving performance on other vendors (GE, Philips) or field strengths unverified.

### Retinal imaging genetics

Traditional retinal imaging-genetics studies rely on expert-defined IDPs such as vessel caliber, optic disc morphology, and macular thickness extracted through specialized software^3^. While successful in identifying genetic associations, these approaches have limitations similar to other imaging modalities, including reliance on segmentation quality and a priori definitions of pathological features. SSL approaches in retinal imaging have focused on leveraging both 2D fundus photography and 3D optical coherence tomography (OCT) to capture broader morphological variation (Table 3). Fundus photography captures a 2D projection of the retinal surface, while OCT provides high-resolution 3D cross-sectional maps, enabling detailed assessment of retinal layers.

iGWAS (Image-based Genome-Wide Association Studies) employed a metric learning approach on retinal fundus photographs using paired left and right eye fundus images^36^. A 2D CNN (Inception V3) was trained on 40,000 EyePACS images (32,000 train/8000 val) using an ArcFace contrastive loss^85^, which minimizes the angular distance between eyes of the same individual while maintaining margins between different individuals. The trained phenotyper was then directly applied to UKBB images without fine-tuning to generate 128-dimensional embeddings for 130,329 images. GWAS on these phenotypes identified 14 independent loci associated with the embeddings directly, including 3 novel loci (*DCT, PDE6G, EPHB4*) linked to retinal structure and function that were missed by traditional color-trait GWAS.

ContIG (Contrastive Imaging and Genetics) applied contrastive learning between retina fundus images and genetic data^55^. Using a ResNet-50 for images and multi-layered perceptrons (MLPs) for genetics, the model was trained to distinguish matched image-genotype pairs from random ones using a batch-wise contrastive loss. The framework integrated multiple genetic modalities, including raw SNPs, polygenic scores (PGS), and burden scores. In an analysis of 155,238 UKBB left and right eye fundus images, ContIG identified phenotypic dimensions that yielded 16–22 independent loci (depending on the genetic modality used), outperforming baseline self-supervised methods (e.g., SimCLR, BYOL) which found only 2–17 loci. Furthermore, the learned features demonstrated superior performance on downstream clinical tasks, such as diabetic retinopathy detection and cardiovascular risk prediction.

Similar to the case for brain images, TransferGWAS applied transfer learning to fundus images using two distinct pretraining strategies on a ResNet-50 architecture: one pretrained on ImageNet (natural objects) and another on EyePACS (retinal pathology). Feature extraction followed by PCA yielded 20 total phenotypes (10 per model)^86^. The study identified 60 independent loci, including 7 novel associations. Interestingly, the phenotypes derived from EyePACS pretraining captured retina-specific pathology, whereas ImageNet-derived phenotypes captured broader morphological features like pigmentation and achieved greater overall discovery power. The interpretation approach involved training separate ResNet18 networks to predict specific genotypes from fundus images, followed by visual inspection of high/low-activating images by experienced ophthalmologists.

Moving to OCT images, Sergouniotis et al. applied an autoencoder-based approach to 2D OCT macular thickness maps^40^. Optical coherence tomography (OCT) provides high-resolution 3D cross-sectional maps of retinal structure, enabling detailed assessment of retinal layer thickness and morphology. The study trained a 2D CNN autoencoder to compress segmented thickness maps into a 64-dimensional latent space, which was further reduced to 25 PCs. Both the original 64-dimensional latent features and the 25 PCs were employed as quantitative imaging phenotypes for genetic association analysis. GWAS on these traits identified 118 significant loci, with 41 achieving independent replication in a validation cohort. Of these, at least 20 loci were novel and not previously associated with retinal phenotypes. To interpret these latent features, the authors utilized retinal thickness difference maps. First, they linked specific latent dimensions to known medical conditions (e.g., non-insulin-dependent diabetes) via statistical association. Once an association was established, they subtracted the average thickness maps of confirmed cases from controls to visualize specific pixel-level morphological changes. This allowed them to pinpoint which anatomical regions were affected by the condition associated with the latent dimension, effectively mapping the genetic findings from that dimension to specific retinal structures. For instance, this approach revealed that diabetes-associated dimensions captured thinning in the paracentral region corresponding to major retinal vessels, confirming the model’s ability to detect subtle neurovascular pathology. The derived phenotypes were shown to improve risk stratification for glaucoma and cardiovascular disease compared to traditional clinical parameters, demonstrating that SSL can extract clinically relevant biomarkers from structural maps.

Retinal SSL studies demonstrate a clear trade-off between domain specificity and discovery power. Models pretrained on domain-specific data (e.g., EyePACS in TransferGWAS) or using specialized inputs (OCT thickness maps) tend to yield clearer structural associations. In contrast, models using generic pretraining (ImageNet) often capture systemic or pigmentation-related traits with potentially broader pleiotropic relevance.

## Challenges and future directions

The field of self-supervised imaging genetics is transitioning from proof-of-concept discovery to a phase of maturation. While early studies have successfully demonstrated that SSL can recover heritable and interpretable phenotypes that can identify novel loci, the next phase of research must address critical bottlenecks in population diversity, methodological rigor, and model interpretability (Box 1).

Box 1 Current challenges and future directions in self-supervised imaging genetics **Challenges**

**Population diversity and generalizability**

- To the best of our knowledge, every SSL imaging GWAS study identified for this Review uses UKBB, a cohort dominated by middle-aged European-ancestry participants.
- Domain shift across scanners, vendors, and protocols outside UKBB data can degrade model performance outside the training cohort.

**Methodological barriers**

- Most SSL pipelines still depend on conventional preprocessing (e.g., skull stripping, registration), introducing pipeline-specific variability.
- High-dimensional latent spaces can dilute statistical power; no consensus exists on the optimal balance between dimensionality and multiple-testing burden.
- Training on 4D modalities (e.g., cardiac cine MRI) is computationally intensive, raising access and carbon-footprint concerns.

**Interpretability and biological meaning**

- SSL-GWAS interpretation is challenging and largely done post hoc: because latent dimensions are learned without anatomical supervision, biological meaning must be inferred after the fact rather than built into the phenotype definition.
- Perturbation-based decoding can smear effects across anatomical boundaries, and saliency maps can saturate.
- Whether learned features reflect reproducible biology versus dataset-specific artifacts remains open.
- Experimental follow-up of SSL-GWAS loci is essentially absent from the literature.

**Future Directions**

- **Diverse cohorts:** Leverage emerging biobanks for cross-ancestry training and validation, including under-represented populations.
- **Benchmarking and accessibility:** Develop standardized benchmarks reporting heritability per latent dimension, replication rates, and compute costs; release pretrained models and pipelines.
- **Emerging architectures:** Vision Transformers for non-local patterns, video transformers and diffusion models for spatiotemporal dynamics, and CLIP-style multimodal foundation models.
- **Newer modalities:** Extend SSL imaging genetics to abdominal MRI, DXA and lung CT.

## Population diversity and generalizability

A primary limitation in imaging genetics is the lack of diversity in data, which undermines the equity of these methods. To the best of our knowledge, every SSL imaging GWAS study identified for genome-wide discovery uses UKBB, a resource dominated by middle-aged participants of European ancestry. This bias limits the generalizability of SSL-derived findings to pediatric, non-European, or under-represented populations. Consequently, replication remains a bottleneck. The scarcity of external cohorts with comparable imaging and genetic scale makes it difficult to validate discoveries. To ensure generalizability and avoid exacerbating health disparities, expansion beyond the UKBB is urgently needed. Promisingly, other large-scale resources are emerging that may help address this gap. For instance, the Qatar Biobank^20^ is combining genetic data with whole-body and brain MRI in a Middle Eastern population, and the German National Cohort (NAKO)^18,19^ has enrolled a large MRI sub-cohort. Leveraging these diverse biobanks for cross-ancestry training and validation will be essential for the next generation of SSL models.

## Methodological and interpretability barriers

For SSL-specific challenges, domain shift poses a particularly acute barrier for self-supervised models, which are often sensitive to acquisition artifacts. Models trained on uniformly collected and processed UKBB data may fail when applied to diverse and heterogeneous clinical scans. A related concern is that SSL models may inadvertently encode non-biological variation such as scanner differences, acquisition artifacts, or ancestry-related morphological differences as latent features, potentially confounding downstream GWAS. Current studies mitigate this by including standard covariates (e.g., age, sex, and genetic principal components) in association models, but systematic evaluation of residual bias in learned representations remains limited. Furthermore, despite the self-supervised label, most SSL pipelines still rely on conventional preprocessing such as skull stripping and registration, which introduces its own variability. Computational burden is another significant constraint. Training models on high-dimensional modalities (e.g., 4D cardiac cine MRI) is resource-intensive. This creates a barrier for smaller and less-well-resourced research groups and raises concerns about the carbon footprint of model development. Simultaneously, the high dimensionality of latent spaces can severely dilute statistical power. While dimensionality reduction (e.g., PCA) helps, there is no consensus on the optimal balance between retaining morphological detail and maintaining statistical efficiency.

Interpretability and biological validation are not a single step. They involve five separate questions, and each needs its own evidence. First, image localization asks which image regions contribute to a latent feature. Saliency maps, occlusion analyses, and decoder-based perturbations answer this, but knowing where a feature comes from does not tell us what it means^37,56^. Second, feature stability asks whether the same feature appears again when the model is retrained or when preprocessing, scanners, or cohorts change. This is difficult because latent dimensions are not ordered in a fixed way, so a dimension from one model does not correspond to the same dimension in another^87,88^. Third, genetic replication asks whether an association holds in an independent sample. It shows the association is real, but not what the phenotype measures. Replication in a held-out split of the same cohort is also weaker than replication in a separate cohort^89^. Fourth, biological mechanisms require further evidence, such as fine-mapping, colocalization with molecular quantitative-trait loci, pathway analyses, or experimental follow-up^90,91^. Fifth, clinical relevance requires association with meaningful traits or outcomes, added value beyond established IDPs, and validation in independent clinical cohorts^92–94^. Most current SSL imaging-genetics studies address only some of them. A latent feature is therefore better described by the specific evidence available for it than by a general label such as “biologically validated.”

Interpretability remains a persistent difficulty even at the first of these levels. While perturbation-based decoding and saliency mapping provide partial insights, they have concrete limitations: decoder perturbations often smear effects across anatomical boundaries rather than being localized, and saliency maps can saturate, obscuring the magnitude of specific features^37,95^. Feature stability is rarely tested directly. Ensembling across many independently trained models, as in the Unsupervised Phenotype Ensemble, reduces the effect of single-model instability but does not measure it^59^. Clinical translation from SSL-discovered loci to therapeutic targets also remains largely theoretical, with experimental follow-up of SSL-GWAS loci essentially absent from the literature.

## Benchmarking and standardization

To usher the field into maturation, we need the development of standardized benchmarks and open evaluation frameworks, which would allow consistent comparison of SSL models across datasets and ensure reproducibility. These benchmarks should explicitly track metrics such as heritability per latent dimension, average replication *p* values for top hits, and compute/energy costs to allow for transparent comparison of SSL architectures. Benchmarks should also test incremental value directly, by computing SSL phenotypes and expert-defined IDPs in the same individuals under matched analytic conditions and reporting loci unique to each and shared between them. Equally important is the release of pretrained models and standardized preprocessing pipelines, which would lower barriers to entry for research groups without extensive computational resources and enable direct comparison of downstream GWAS results across studies. Recent work has emphasized systematic benchmarks; for example, EmbedGEM evaluates whether embeddings are both genetically informative and clinically meaningful^96^.

## Emerging architectures

The next frontier of SSL involves moving beyond static CNNs to architectures that better capture global context and temporal dynamics. Vision Transformers (ViTs)^97,98^ are emerging as a powerful alternative to traditional convolutional approaches, leveraging attention mechanisms to capture non-local patterns (such as hemispheric symmetry in the brain) that local receptive fields may miss. Foundation model approaches inspired by vision-language pretraining (e.g., CLIP-style contrastive frameworks)^73^ are also being adapted to imaging-genetics, though current implementations such as COMICAL operate on pre-extracted IDPs and pre-selected variants rather than learning representations from raw images^99^. Simultaneously, the scope of SSL is expanding beyond structural T1-weighted imaging to include microstructural modalities, such as modeling white matter integrity via diffusion MRI.

In cardiac imaging, the focus is shifting toward spatiotemporal modeling. Newer architectures, including diffusion models^100^ and video transformers^101^, are being designed to explicitly encode temporal dynamics rather than treating frames as static independent channels. These methods aim to capture complex physiological phenotypes, such as rhythmic motion and myocardial strain. Rigorous validation of these advanced architectures in independent, diverse cohorts remains a priority to confirm their clinical utility over established CNN-based baselines.

Beyond architectural advances, extending SSL imaging genetics to currently underexplored modalities such as abdominal MRI^102,103^, DXA^104,105^, lung CT^106,107^, and histopathology^108^ where supervised methods have already demonstrated substantial heritable signal represents a near-term opportunity to expand the scope of genetic discovery. For example, HistoGWAS recently demonstrated that pretrained histology encoders can be used to derive quantitative tissue embeddings for GWAS, showing histopathology as an emerging direction for image-based GWAS beyond conventional radiological imaging^108^.

## Conclusion

Self-supervised deep learning approaches allow scalable derivation of complex, high-dimensional, and heritable imaging phenotypes by enabling the data itself to define rich and reproducible features. Applied across brain, cardiac, retinal, and physiological modalities, these methods have uncovered genetic associations missed by traditional expert-defined traits and in some cases demonstrated greater heritability. Important challenges remain, including limited interpretability, lack of standardized pipelines, high computational demands, and restricted reproducibility and generalizability beyond largely European ancestry cohorts. SSL-derived phenotypes are therefore best regarded at present as promising complementary tools rather than established, clinically actionable biomarkers. With careful validation and broader application, SSL-derived phenotypes have the potential to advance genetic discovery and translate into meaningful improvements in human health.

## Supplementary information

Transparent Peer Review file

## Data Availability

No datasets were generated or analysed. All studies discussed are published and are cited in the reference list.
