# Supplementary material for: A review of self-supervised imaging phenotypes for genome-wide association studies
Source: Commun Biol. 2026 Oct 23;9:1394. doi: 10.1038/s42003-026-11199-4 (PMC13694269; doi:10.1038/s42003-026-11199-4)
Supplement: Supplementary file 1 [file 42003_2026_11199_MOESM1_ESM.pdf]

nature portfolio

Peer Review File

A Review of Self-Supervised Imaging Phenotypes for Genome-Wide Association Studies

Corresponding Author: Professor Degui Zhi

This file contains all reviewer reports in order by version, followed by all author rebuttals in order by version.

Version 0:

Reviewer comments:

Reviewer #1

(Remarks to the Author)
This manuscript reviews the state of AI and self-supervised learning (SSL) derived image-derived phenotypes (IDPs) and their link with GWAS with particular focus of applications in brain MRI, cardiac MRI, and retinal imaging. Although, this is a review, it is very difficult to make that out from the title. As it stands, the title seems to be indicative of a SSL-derived IDP method for GWAS. I think the title should be revised to indicate the actual research contribution.

There are several concerns related to how the review is structured and presented. The content in most places reads fine but does not read like a deep review of the field but rather than just a collection of methods/techniques followed by some arbitrarily chosen applications. A systematic review needs to be broad and explain the field in a more deeper sense so that the readers can gauge the current status as well as leave with their own ideas of methods or application areas. This review hits neither of those objectives. Below I will lay out some of my major concerns which follows this meta-review.

1. In the overall framework section, most of the standard approaches are very subjectively written. Comments like "validation sets should have a sufficient number of images for model training (typically thousands)" and similar sentences are hand-wavy. The authors should perhaps cite papers which have done deeper analysis of sample-size driven power of SSL learning in the field and give concrete numbers.

2. GWAS of imaging traits has been a pretty long history which the introduction section does not allude to and the Phase B section mainly describes a standard GWAS pipeline and quotes tools such as BOLT-LMM and fastGWA which are standard GWAS methods (but there are many many others who can handle similar tasks). So, overall this seems to be a patched together section. Similar arguments flow for Model Interpretation section.

Overall, this section feels like an introduction to the further sections but not written as an introduction at all. I would advise the authors to restructure it to reflect more of an integrated introduction with more cohesiveness. If you want to remark about a particular method, that is okay, as long as it serves a broader purpose.

3. The reference list in the SSL approaches subsection, as it often happens with reviews is never complete or comprehensive, which is understandble.
While the section provides a high-level categorization of approaches such as reconstruction, contrastive, redundancy reduction, and multimodal alignment it remains largely descriptive. But, I think these sections are missing very important references and methods, which should definitely be included.

In particular, it lacks a unifying perspective linking these methods as alternative strategies for learning invariant representations while avoiding collapse, and does not explain the underlying mechanisms that enable different approaches (e.g., negative sampling in contrastive learning or decorrelation in redundancy reduction). Important methods such as BYOL, VICReg, MoCo, and especially CLIP in the multimodal setting are absent, and there is no discussion of critical design factors like data augmentations or known limitations such as false negatives, large batch requirements, and sensitivity to data regimes. Further, the section does not connect these paradigms to downstream performance or provide even minimal mathematical explanations, which limits its depth and completeness as a review.

4. In brain imaging genetics section, these issues remain and is a feature of the manuscript as a whole. Again some glaring omissions have to do with the lack of inclusion of methods which uses CLIP-like encoders for doing imaging-derived GWAS
a. Machado Reyes, D., Burch, M., Parida, L., & Bose, A. (2025). A foundation model for learning genetic associations from brain imaging phenotypes. Bioinformatics Advances, 5(1), vbaf196.
b. Chen, H., Liao, Y., Tang, L., Wei, X., Li, T., & Chen, W. (2025). Multivariate genome-wide analysis reveals shared genetic architecture and brain structural correlates of human cognitive abilities. Scientific Reports, 15(1), 41596.
.. and many others like this.

5. There is a lack of comparison across methods and also lacking is a unifying framework which merges the reconstruction and contrastive learning-like approaches. Also missing are methodological gaps like sensitivity to multiple distributions in the cohort, bias, etc. But perhaps the most glaring omission is the lack of theoretical grounding and mathematical explanations.

6. The applications ares although discussed in some detail seem to be arbitrary.

Minor concerns:

1. Check formatting of the tables
2. Use subsection headers to delineate between sections in the applications

In summary, the manuscript addresses an important and timely topic at the intersection of self-supervised learning and imaging genetics. However, in its current form, the methodological discussion remains largely descriptive and lacks the depth expected of a review in this area. Key objectives (e.g., contrastive losses, redundancy reduction criteria, or mutual information formulations) are not explicitly defined, and there is little discussion of the principles that prevent representation bottlenecks or govern the trade-offs between competing approaches (e.g., invariance vs information preservation, interpretability vs predictive power). As a result, the presentation reads more like a catalog of methods than a critical synthesis of the field. This lack of analytical depth makes it difficult to assess when and why one class of methods should be preferred over another, particularly in the context of imaging genetics.

For a review article, it is essential to go beyond surface-level descriptions and provide conceptual clarity, mathematical grounding, and critical evaluation. I strongly encourage the authors to include formal objective functions wherever appropriate, articulate underlying mechanisms and trande-offs, and synthesize literature into a coherent framework with rigor and depth. Perhaps, the authors can use an unsupervised approach to identify key manuscripts in this intersection area from PubMed and perform BERT-like topic modeling to observe different key areas and major contributions.

Reviewer #2

(Remarks to the Author)
The review from Patel K and colleagues outlines methods and analytical frameworks to use imaging data as outcomes in genome wide association studies.

A general comment is that the text is written as a narrative review where different methodologies are described but not fully exploited and a model for integration of large datasets (images and genetic variations) is proposed. This is true for the derivation of the quantitative phenotypes from images as well as for GWAS analyses. Similar attempts have been done using different types of phenotypes and the novelty of the approach proposed does not come forward.

The tables are impossible to read, they lack references and a clear context. Do they refer to methods the Authors or other have developed?

In which way reconstruction of images, and the genetic analyses of it should translate in progress?
We use already measures that are derived from images, as an example calcium score for intimal calcium, but we use it because it represent a specific stage of the disease. In which way the transformations described can be applied to improve our understanding of a disease/process?

Reviewer #3

(Remarks to the Author)
I co-reviewed this manuscript with one of the reviewers who provided the listed reports. This is part of the Communications Biology initiative to facilitate training in peer review and to provide appropriate recognition for Early Career Researchers who co-review manuscripts.

Reviewer #4

(Remarks to the Author)

This manuscript provides a clear and well-structured overview of recent developments in self-supervised learning approaches for imaging genetics. The review is informative and scientific sound.
However, a few minor issues could be addressed to improve the the manuscript.

The manuscript states that a literature search was conducted, but the criteria used to select the studies included in the review are not described. Providing a brief description of the search strategy (e.g., databases consulted, keywords, or general inclusion criteria) would improve transparency and help readers better understand how the reviewed studies were identified.

Some statements comparing performance across studies appear somewhat definitive. For example, the manuscript notes that Genetic InfoMax "achieved the highest heritability among current approaches." Given that the reviewed studies differ in preprocessing pipelines, phenotype definitions, and GWAS sample sizes—as also acknowledged later in the text—such cross-study comparisons may be difficult to interpret directly. It may therefore be preferable to use slightly more cautious wording (e.g., "reported higher heritability" or "among the highest reported") to avoid implying a direct methodological ranking.

Reviewer #5

(Remarks to the Author)
The authors provide a review of self-supervised learning (SSL) approaches for extracting AI-derived endophenotypes from medical imaging data. The manuscript introduces the general framework of SSL methods, followed by discussions on downstream GWAS and model interpretation strategies. In addition, the authors summarize several organ-specific applications of SSL in imaging genetics, including studies of the brain, heart, and retina, and highlight key findings regarding model architectures and genetic discoveries. Finally, the review outlines current challenges and future directions in SSL-based imaging genetics, such as limited dataset availability, European ancestry bias, and methodological and interpretability barriers. Overall, this manuscript provides a useful overview of the emerging field of SSL-based imaging genetics. However, before publication, I have some comments and suggestions that may help improve the clarity and comprehensiveness of the review.

In the Introduction, the authors present a rather critical perspective on expert-defined imaging-derived phenotypes (IDPs) while emphasizing the advantages of SSL-derived phenotypes. While it is true that expert-defined IDPs have limitations, such as sensitivity to image quality and reliance on existing anatomical knowledge, they have also played a crucial role in many successful imaging-genetics studies. It would therefore be helpful if the authors could provide a more systematic comparison between expert-defined IDPs and SSL-derived phenotypes. For example, discussing their respective strengths, limitations, and suitable application scenarios would help readers better understand the practical advantages of SSL approaches.

In the second paragraph of the Introduction and in the Discussion section, the authors briefly mention several imaging cohorts and biobanks other than the UKB, and the review primarily focuses on studies based on UKB data. Although these additional datasets are generally smaller in scale, it would still be valuable for the authors to summarize the main findings from studies using these cohorts. Providing a brief overview of their characteristics, strengths, and limitations could offer useful insights and potentially guide future study designs for other cohorts and emerging imaging-genetics projects.

In Lines 130–133, the authors mention that PCA can be used as an efficient dimensionality reduction method when the number of latent features is large. While PCA is indeed widely used, it would be beneficial to discuss additional strategies for summarizing or integrating latent representations, as this step is crucial for downstream analyses and can also help identify biologically meaningful patterns. For example,
Yang, Z., Wen, J., Erus, G. et al. Brain aging patterns in a large and diverse cohort of 49,482 individuals. Nat Med 30, 3015–3026 (2024).

They proposed R-indices to summarize brain aging patterns across multiple anatomical regions.
The authors discuss SSL applications in three major organ systems (brain, cardiac, and retinal imaging), but I believe the discussion is not comprehensive enough. For these three organ-specific applications, there appears to be only 2-3 pieces of relevant evidence for each organ. In addition, the manuscript briefly mentions other organ systems (e.g., skeletal imaging), but these are not discussed in detail. I am aware that the available literature is limited, the authors could include a short paragraph summarizing emerging applications in other organ systems.

Given the complexity of SSL models, the review could also address issues related to reproducibility, including the availability of pretrained models, standardized preprocessing pipelines, and open benchmarking datasets for imaging-genetics studies. These aspects may represent important future directions for the development and broader adoption of SSL-based approaches.

Version 1:

Reviewer comments:

Reviewer #1

(Remarks to the Author)
I thank the authors for revising the manuscript substantially. However, several of my previous concerns remain along with some fresh concerns.

1. With the inclusion of the clear statement of the inclusion criteria, I think the motivation to study SSL directly applide to raw or minimally-preprocessed images and learned representation based GWAS is a bit too narrow of a focus. On top of this, I also think it is under-motivated. I think for a review in Comms Bio, the introduction should clearly establish the biological problem and give necessary motivation for the problem and then argue that SSL on images along with GWAS is a route that is often taken and hence needed to be reviewed.

The authors should distinguish more clearly between the topics they discuss: (a) SSL as a representation learning objective (b) medical image phenotyping, and (c) GWAS on image-derived traits. The present introduction quickly arrives at the inclusion criteria before laying out the biological motivation.

2. I think the authors should refrain from statements like "SSL methods rely on supervised loss functions" as this is not precise. SSL often uses supervised-style optimization objectives or pseudo-label tasks but not explicit phenotype/trait labels. These nuances should be mentioned and phrased carefully.

3. Balance the Table 1 in more realistic/practical terms. The table frames expert-defined IDP as too rigid and limited. Many SSL algorithms still use preprocessing choices, organ/ROI selection, etc. architecture designs which learns with some form of anatomical prior. So, saying "SSL-derived phenotypes learn without anatomical prior" is too strong. The table should also acknowledge that SSL embeddings can encode non-biological variation such as batch effects, demographic structure, or image artifacts. As this review targets genetic discovery, the table should explicitly compare the two phenotype classes in terms of post-GWAS interpretability, burden tests, and biological validation.

4. The field of SSL-based brain imaging/cardiac/retinal genetics is emerging, but the evidence base summarized here remains small and highly heterogeneous. The manuscript needs to articulate why this nascent field requires a standalone review rather than a section within a broader review of imaging genetics, SSL-derived medical-image phenotypes, or deep-learning-based GWAS phenotyping. As written, the motivation rests heavily on a small set of studies, while the synthesis does not yet extract sufficiently general methodological or biological lessons from them.

5. The tables indicating how many loci were found is informative but it would be good to see if the learned phenotypes are reproducible, nonredundant (compared with established IDPs), biologically interpretable, clinically relevant (variant effects can be studied), etc.

6. There are numerous issues with the language (copy editing), missing commas, the tables are not well-wrapped and difficult to follow. The organization of the tables need clear improvement.

7. I think it would greatly benefit the field if the authors include a decision map as a main takeaway of their review. This table can serve as a summarized, one-stop place to allow people with relevant biological tasks decide on which SSL methods to choose, what their (dis)advantages are, etc.

Reviewer #2

(Remarks to the Author)
I think the Authors have addressed the comments raised and the review reads better now.

Reviewer #4

(Remarks to the Author)
Thank you for the revised manuscript and detailed responses. I have reviewed the revised version and am satisfied that my previous concerns have been adequately addressed. I have no further major concerns and support acceptance.

Reviewer #5

(Remarks to the Author)
The manuscript has improved substantially in response to the previous reviews. The authors have clarified the relationship between self-supervised learning (SSL) and expert-defined imaging-derived phenotypes, expanded the historical and methodological context, simplified the tables, improved the figures, strengthened discussion of limitations, and removed AI-generated visual elements.

Overall, the authors have made a meaningful effort to address the previous comments, and the manuscript has clear potential. However, further revision is required to clarify the methodological scope, strengthen the critical synthesis, and ensure that claims about genetic discovery, reproducibility, and biological interpretation are appropriately calibrated. Some remaining comments are below:

Scope and terminology require further clarification. The manuscript uses “SSL” broadly, but some included examples appear

to involve unsupervised learning, transfer learning, supervised models, or genotype-informed multimodal representation learning. The authors should either apply a stricter SSL definition or broaden the scope and clearly classify each approach.

Potential circularity in genotype-informed embeddings needs explicit discussion. For approaches that use genotype data during representation learning and subsequently perform GWAS, the manuscript should discuss data leakage, sample overlap, and the need for non-overlapping training and association-testing datasets.

The comparison between expert-defined IDPs and SSL-derived phenotypes remains too categorical. Table 1 is useful but should better acknowledge that both approaches depend on preprocessing, technical choices, and cohort characteristics. SSL approaches do not necessarily operate without anatomical assumptions, and expert-defined phenotypes are not uniformly more reproducible.

The review would benefit from stronger critical synthesis. Rather than primarily listing architectures and applications, the manuscript should more directly address when SSL-derived features provide information beyond conventional IDPs, how this should be evaluated, and what evidence is needed for biological or clinical interpretation.

Interpretability and biological validation should be discussed more rigorously. Heritability, saliency maps, or association signals alone do not establish that a latent feature is a biologically meaningful phenotype. The authors should distinguish image localization, feature stability, genetic replication, biological mechanism, and clinical relevance.

The literature-search strategy should be more transparent. Even as a narrative review, the manuscript should provide the search date, databases, representative search terms, inclusion/exclusion criteria, and treatment of preprints. The literature should be updated and bibliographic details checked. Recent relevant studies should be included where appropriate, and preprints should be clearly labelled or replaced by peer-reviewed publications when available.

The conclusions should be moderated. The evidence base remains relatively small, predominantly based on UK Biobank data, and has limited cross-cohort replication or functional validation. The conclusion should present SSL-derived phenotypes as promising complementary tools rather than established, reproducible, or clinically actionable biomarkers.

Version 2:

Reviewer comments:

Reviewer #1

(Remarks to the Author)
I thank the authors for incorporating all the proposed changes in the manuscript. I would just add a couple of more things for the authors to look into for SSL in image-derived phenotype GWAS:

1. Highlighting the methods for generalizability. There is a small but growing literature on this. Such as a 2026 preprint, which explicitly examined the replicability of unsupervised deep-learning image phenotypes across random initialization, data splits, and independent UK Biobank and ADNI training cohorts, and further assessed whether this stability propagated to GWAS discoveries. I think this and related work can strengthen this paper.

2. Polygenic risk score is a growing literature in SSL. Papers like GEMCONT have appeared Sens, D., Shilova, L., Dalca, A. V., Schnabel, J. A., & Casale, F. P. (2026, May). GEMCONT: Genetics-based Multimodal Contrastive Learning Enhances Phenotypic embeddings and Boosts Genetic Discovery. In 9th International Conference on Medical Imaging with Deep Learning, MIDL 2026 (pp. 3443-3463), uses genetic supervision specifically to learn disease-relevant phenotypic embeddings. If PRS are incorporated here, it would strengthen the material substantially for representation learning and downstream genetic analysis.

Reviewer #5

(Remarks to the Author)
The manuscript has improved appreciably after the previous revision. In particular, the clearer biological motivation, the discussion of genotype-informed circularity, the more balanced Table 1, the new decision map, and the distinction among localization, feature stability, genetic replication, biological mechanism and clinical relevance. The authors have also added language stating that SSL-derived phenotypes should currently be regarded as complementary tools rather than clinically actionable biomarkers.

Nevertheless, several minor issues remain and require further revision.

Check loci and leading SNPs, eg the Table 4 has a column headed “Loci (discovery/replication)” and reports “300/182”. The number 182 is not a number of replication loci; it is the number of replicated lead SNPs.
Similarly for “93 joint, 86 ECG, 33 MRI” under the heading “Loci”. Yet the manuscript's own narrative explicitly describes these as lead SNPs, not independent loci.
Please replace “Loci” with the appropriate signal type for this row, or redesign the table so that “lead SNPs” and “loci” cannot

be inadvertently compared as equivalent quantities.

Search cutoff is internally inconsistent with the literature actually included, eg the Methods/Introduction states:
“We searched PubMed and Google Scholar through May 2026...”. Yet the manuscript cites and discusses the Zhao et al. UDIP-FA article, whose final version was published on 3 June 2026.

Open Access This Peer Review File is licensed under a Creative Commons Attribution 4.0 International License, which permits use, sharing, adaptation, distribution and reproduction in any medium or format, as long as you give appropriate credit to the original author(s) and the source, provide a link to the Creative Commons license, and indicate if changes were made.
In cases where reviewers are anonymous, credit should be given to 'Anonymous Referee' and the source.
The images or other third party material in this Peer Review File are included in the article’s Creative Commons license, unless indicated otherwise in a credit line to the material. If material is not included in the article’s Creative Commons license and your intended use is not permitted by statutory regulation or exceeds the permitted use, you will need to obtain permission directly from the copyright holder.
To view a copy of this license, visit https://creativecommons.org/licenses/by/4.0/

Dear Dr. Inglis,

Thank you for the opportunity to revise our manuscript and for your helpful feedback. We have addressed all of the comments below, attached revised manuscript and manuscript with marked changes.

Editor Comments

(1) Expanding the introduction to comment on the history of imaging genetics research (Reviewer #1) and the use of expert- vs. SSL-defined phenotypes (Reviewer #5).

Response.

Thank you for your suggestion. We have expanded the introduction in two ways:

First, we added a new paragraph (paragraph 2) summarizing the history of imaging genetics, tracing the field from early candidate-gene studies (e.g., *APOE ε4, 5-HTTLPR, BDNF Val66Met*) through the replication challenges that highlighted the polygenic architecture of imaging traits, to the large-scale consortium efforts (ENIGMA, CHARGE) that yielded the first robustly replicated loci for brain imaging phenotypes, and finally to the current era of population-scale biobank studies (**Line number 38-52)**.

Second, we added a direct comparison of expert-defined IDPs and SSL-derived phenotypes in the SSL paragraph. We frame SSL not as a replacement for expert-defined approaches but as a means of expanding the phenotypic space. We have also now included a new table "**Table 1. Expert-Defined IDPs vs. SSL-Derived Phenotypes**" (**Line number 140**) and revised some lines in introduction to more clearly distinguish between the two approaches (**Line number 89-111).**

(2) Providing additional context on method performance (Reviewers #1 and #4), what is considered as "SSL" (Reviewers #2-3), and how the method used to reconstruct images relates to the genetic analysis (Reviewers #2-3).

Response. We have addressed these points with three targeted revisions.

Definition of SSL. We now clarify in introduction (**Line number 113-119**).

"Although SSL is a subclass of unsupervised learning, SSL methods rely on supervised loss functions which distinguish them from traditional unsupervised learning such as cluster, dimension reduction, and density estimation. Although autoencoders were invented before the term SSL got popular, they are often categorized as an SSL because that the idea of deriving a compact representation (bottleneck) and using the decoder as a guide to train the encoder are very much in line with the philosophy of SSL."

Image reconstruction and genetic analysis. We revised the "Self-Supervised Learning Approaches: reconstruction-based methods" section to explicitly state that the goal is not the reconstructed image but the compressed latent representation, which serves as the quantitative phenotype for GWAS. (**Line number 260-269**)

Method performance. We softened performance-related claims throughout the manuscript to avoid implying direct methodological rankings across studies with different preprocessing pipelines, phenotype definitions, latent dimensionality, GWAS sample sizes, replication designs, and multiple-testing burdens. We removed claims that any method achieved the "highest" heritability and removed mean heritability as a table-level comparison

parameter. We also added or strengthened cautionary language in the Abstract ("Early studies suggest that these data-driven approaches can complement traditional IDPs and may expand the phenotypic space available for discovery of heritable imaging traits, although broader validation across cohorts, ancestries, scanners, and imaging protocols remains needed."), Introduction ("While challenges remain in reproducibility, cross-cohort generalization, interpretability, and biological validation, SSL-derived phenotypes represent a promising complement to expert-defined IDPs and may scale to biobank-level analyses; however, their broader adoption will require standardized benchmarking, external replication, and validation across diverse biobank and clinical cohorts."), individual sections, Challenges and Future Directions section, and Box 1. We wanted to emphasize that early studies suggest SSL-derived phenotypes may complement traditional IDPs, but that broader validation across cohorts, ancestries, scanners, imaging protocols, and clinical settings remains needed.

(3) Commenting (where possible) on emerging applications to other imaging modalities (Reviewer #5), the use of cohorts other than the UKBB (or lack thereof; per Reviewers #2-3 and Reviewer #5), and more generally the limitations faced by researchers in this field (Reviewers #2-3: points regarding generalizability and interpretability of learned features).

Response. Thank you for your comment.
Emerging applications to other imaging modalities (Reviewer #5). We have expanded the discussion of emerging modalities in two ways. First, we added a recently published diffusion MRI study to the Brain Imaging Genetics section and Table 3 because it falls directly within the scope of the review: it applies an unsupervised 3D convolutional autoencoder to voxel-wise fractional anisotropy maps, derives UDIP-FA embeddings, and uses these embeddings as quantitative phenotypes for multistage multivariate GWAS. Second, we added a sentence to the Future Directions section identifying abdominal MRI, DXA, lung CT, and histopathology as underexplored modalities where traditional imaging GWAS or AI-enabled phenotyping studies have already demonstrated substantial heritable signal. We also added HistoGWAS as an emerging example of image-based genetic discovery beyond conventional radiological imaging. HistoGWAS performs GWAS using AI-derived histology embeddings as quantitative phenotypes, but because it focuses on histopathology rather than MRI, fundus photography, OCT, or diffusion MRI, and because it represents an adjacent tissue-imaging application rather than a mature organ-specific radiological imaging literature, we discuss it in Future Directions rather than creating a separate section or adding it to the organ-specific tables.

Limitations regarding generalizability and interpretability (Reviewers #2–3). We thank the reviewers for highlighting the need to more clearly discuss the limitations of SSL-based imaging genetics. In the Methodological and Interpretability Barriers subsection, we further added that SSL models may inadvertently encode non-biological variation, including scanner differences, acquisition artifacts, or ancestry-related morphological variation, as latent features. We now note that although current studies typically adjust for standard covariates such as age, sex, and genetic principal components, systematic evaluation of residual bias in learned SSL representations remains limited. Finally, we added that clinical translation from SSL-discovered loci to therapeutic targets remains largely theoretical, with experimental follow-up of SSL-GWAS loci essentially absent from the current literature.
(Line number 677-692)

(4) From an editorial perspective, we would also note:

(i) You mention that some icons/figures were made using Google Gemini. Per our artificial intelligence policy (https://www.nature.com/nature-portfolio/editorial-policies/ai#generative-ai-images), we generally cannot allow for the use of AI-derived images, and would ask that you replace these with copyright-free icons from Biorender or other databases (or, redraw images yourselves).

Response. Thank you for your comment. We have removed all AI-generated icons used in the figures from the manuscript. All images now created in Biorender using the icons available in biorender, cardiac MRI example images from the UK Biobank, and brain MRI visualizations generated from the MNI152 standard-space template (Fonov et al., 2011). The figures no longer contain any AI-derived visual content. We have added a line UK Biobank images in this figure are reproduced by kind permission of UK Biobank ©.

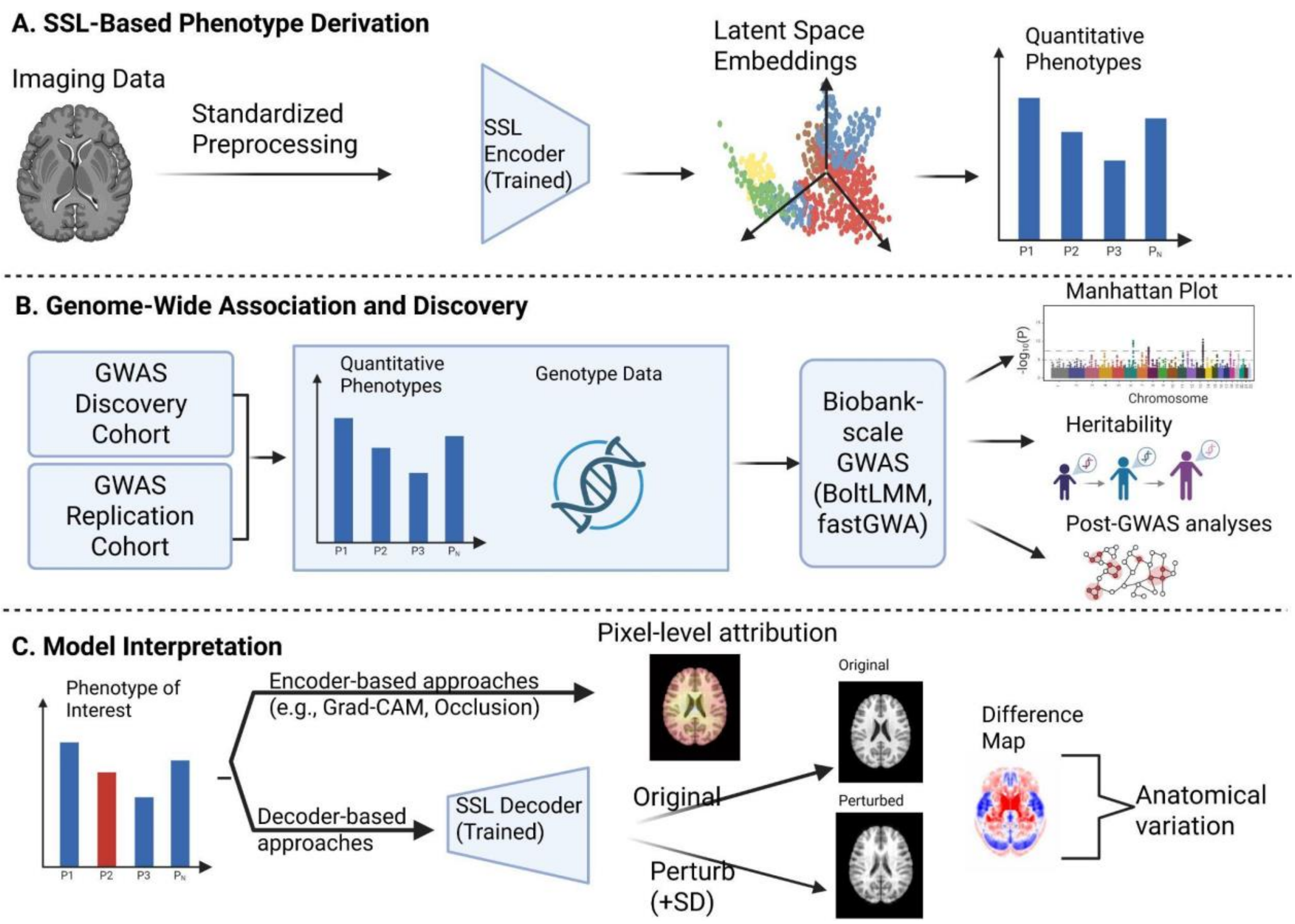

Figure 1

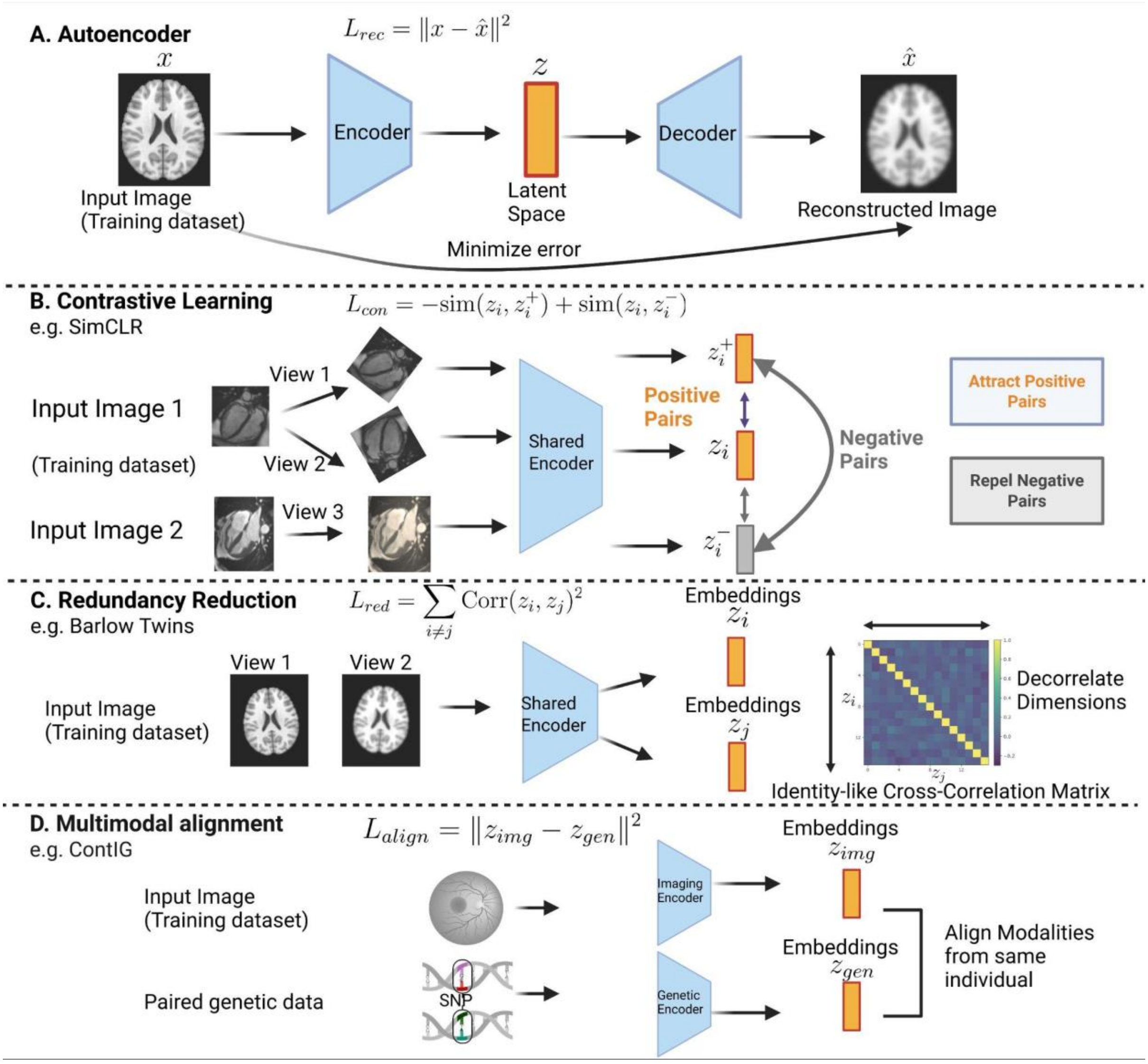

Figure 2

(ii) Fig 1b: The arrows make it look like GWAS discovery & GWAS replication respectively only consider quantitative phenotypes vs genotype data. Please consider merging the arrows, to avoid any confusion.

Response. Thank you for your comment. We have merged the arrows in Figure 1B so that both the GWAS Discovery Cohort and GWAS Replication Cohort now feed into a single combined block containing both quantitative phenotypes and genotype data. This clarifies that both cohorts contribute both data types to the biobank-scale GWAS analysis.

(iii) Fig 2a: Put the "A" label to the leftmost side, for consistency with other subpanels

Response. Thank you for your comment. We have moved the "A" label to the leftmost side of the panel, consistent with the labeling of panels B, C, and D.

(iv) Figg 2b: Per our comments on the last draft of the Review, It’s still unclear what the vector bar plot is meant to represent. Could this be depicted instead as examples of imaging data, as a visual (e.g. examples of positive vs negative pairs)?

Earlier comment: For Fig 2b1, it’s unclear what the vector bar plot is meant to represent. Could this be depicted instead as examples of imaging data, as a visual (e.g. examples of positive vs negative pairs)? In any case, please consider changing the color scheme to avoid red-green color comparisons (for any colorblind readers)

Response. Thank you for your comment. We have now updated the figure to clearly show the latent space representation from augmented views of the same image while repelling representations from different images. Additionally, we have updated the color scheme from red-green to blue-orange throughout the panel to ensure accessibility for colorblind readers.

(v) In the previous draft, there was a statement of "The higher number of loci reported by Rakowski et al." that has now been changed to "The higher number of loci reported by them" (Line 319). Please be sure to include a citation for this statement and appropriately discuss Rakowski et al (ref #38?)

Response. Thank you for your comment. We have now added the citation.

(vi) Per our earlier comments (and the remarks from Reviewers #2-3), it seems like the Tables may still be too granular (with details like interpretability, latent space, etc) for more generalist readers to easily understand. We would still encourage you to simplify these tables, or represent some of the details in a complementary figure. In your previous response, you mentioned that these concepts were broadly visualized in Fig 1-2 (at least, for Table 1), but it is unclear how specific parameters like latent space are defined in those display items.

Previous comment: I think it’s nice to have this kind of summary table, but I worry (similar to notes above) that some of the details (interpretability, latent space, etc) would be too granular for most readers. Would it be possible to have a visual representation of the differences between these approaches as a figure? (Either as a complement or replacement to this Table).

Response. Thank you for your comment. We have simplified all three tables (Now renumbered as Tables 3-5) to improve readability for a generalist audience. Specifically, we removed the following columns from each table: Preprocessing, Latent Space, Mean h^2, and Interpretability. Our rationale for each removal is as follows:

Preprocessing and Latent Space: These are technical parameters that vary by study-specific implementation choices. The general concepts of preprocessing and latent-space compression are already visualized in Figures 1 and 2.

Mean heritability: In line with Reviewer #4's concern that cross-study heritability comparisons are difficult to interpret given differences in preprocessing pipelines, phenotype definitions, and GWAS sample sizes, we have removed this column to avoid implying a direct methodological ranking. Heritability is now discussed qualitatively in the main text where appropriate context exists.

Interpretability: The entries in this column required long, jargon-dense phrases that were difficult to parse at a glance. Each study's interpretability strategy is now described in the main text, where sufficient context can be provided.

We additionally consolidated the **N (Train / Val / Discovery / Replication)** column into a simpler Discovery / Replication N column, since train/validation sizes are model-development details that are not central to interpreting GWAS findings.

We added a Reference to each study row (per Reviewer #2's request for clearer citation context) and unified column structure across all three tables.

The simplified tables now retain only the columns most relevant for a generalist comparison: study (with reference), imaging modality, SSL approach, architecture, discovery/replication cohort sizes, loci identified, and novel loci. This reduces each table to six to seven columns of concise entries while preserving the key comparative information.

(vii) Per the remarks from Reviewers #3 and #5 about limitations, please consider including a Box summarizing current challenges facing this field (along with future directions). Please keep in mind that the final piece is restricted to 8 total display items (tables, figures, and/or boxes), and limited to ~8k words.

Response. Thank you for your comment. The Challenges section groups current limitations under three themes: (i) population diversity and generalizability, noting that every SSL imaging GWAS study within our literature cutoff uses UK Biobank; (ii) methodological barriers, covering preprocessing dependence, the dimensionality-power trade-off, and computational cost; and (iii) interpretability and biological meaning, directly addressing Reviewer #3's concern by explicitly stating that SSL-GWAS interpretation is challenging and largely post hoc, and noting the near-absence of experimental follow-up of SSL-discovered loci. The Future Directions section highlights priorities including diverse cohort expansion, standardized benchmarking, emerging architectures (Vision Transformers, video/diffusion models, CLIP-style multimodal foundation models), and extension to underexplored modalities (abdominal MRI, DXA, lung CT, histopathology). We now have added the Box 1.

Box 1 | Current challenges and future directions in self-supervised imaging genetics
Challenges
Population diversity and generalizability

- To the best of our knowledge, every SSL imaging GWAS study identified for this Review uses UKBB, a cohort dominated by middle-aged European-ancestry participants.
- Domain shift across scanners, vendors, and protocols outside UKBB data can degrade model performance outside the training cohort.

Methodological barriers

- Most SSL pipelines still depend on conventional preprocessing (e.g., skull stripping, registration), introducing pipeline-specific variability.
- High-dimensional latent spaces can dilute statistical power; no consensus exists on the optimal balance between dimensionality and multiple-testing burden.
- Training on 4D modalities (e.g., cardiac cine MRI) is computationally intensive, raising access and carbon-footprint concerns.

Interpretability and biological meaning

- SSL-GWAS interpretation is challenging and largely done post hoc: because latent dimensions are learned without anatomical supervision, biological meaning must be inferred after the fact rather than built into the phenotype definition.
- Perturbation-based decoding can smear effects across anatomical boundaries, and saliency maps can saturate.
- Whether learned features reflect reproducible biology versus dataset-specific artifacts remains open.
- Experimental follow-up of SSL-GWAS loci is essentially absent from the literature.

Future Directions

- **Diverse cohorts:** Leverage emerging biobanks for cross-ancestry training and validation, including under-represented populations.

- **Benchmarking and accessibility:** Develop standardized benchmarks reporting heritability per latent dimension, replication rates, and compute costs; release pretrained models and pipelines.
- **Emerging architectures:** Vision Transformers for non-local patterns, video transformers and diffusion models for spatiotemporal dynamics, and CLIP-style multimodal foundation models.
- **Newer modalities:** Extend SSL imaging genetics to abdominal MRI, DXA and lung CT.

Reviewer #1 (Remarks to the Author):

a) This manuscript reviews the state of AI and self-supervised learning (SSL) derived image-derived phenotypes (IDPs) and their link with GWAS with particular focus of applications in brain MRI, cardiac MRI, and retinal imaging. Although, this is a review, it is very difficult to make that out from the title. As it stands, the title seems to be indicative of a SSL-derived IDP method for GWAS. I think the title should be revised to indicate the actual research contribution.

Response. Thank you for your comment. Based on editorial suggestions, we have updated title to **"A Review of Self-Supervised Imaging Phenotypes for Genome-Wide Association Studies in Biobanks".**

b) There are several concerns related to how the review is structured and presented. The content in most places reads fine but does not read like a deep review of the field but rather than just a collection of methods/techniques followed by some arbitrarily chosen applications. A systematic review needs to be broad and explain the field in a more deeper sense so that the readers can gauge the current status as well as leave with their own ideas of methods or application areas. This review hits neither of those objectives. Below I will lay out some of my major concerns which follows this meta-review.

1. In the overall framework section, most of the standard approaches are very subjectively written. Comments like "validation sets should have a sufficient number of images for model training (typically thousands)" and similar sentences are hand-wavy. The authors should perhaps cite papers which have done deeper analysis of sample-size driven power of SSL learning in the field and give concrete numbers.

Response. Thank you for your comment. We would like to clarify that our manuscript is a narrative review rather than a systematic review. Our goal is to provide a methodological and biological framework for understanding how SSL-derived imaging phenotypes support genetic discovery, rather than to systematically catalog all methods in the field.

To make this explicit, we have added the following sentence to the Introduction: "*The Review that aims to provide a methodological and biological framework for understanding how SSL-derived imaging phenotypes support genetic discovery.*"

Regarding the specific concern about imprecise language, we agree that the phrase "typically thousands" was vague. We have revised the sentence to ground it in the concrete sample sizes used across the reviewed studies. The revised text now reads: *"Four datasets support an SSL imaging-genetics project: training and validation sets for model development, and discovery and replication sets (with paired imaging and genetic data) for GWAS. Training sets in reviewed studies range from ~2,500 (Sergouniotis et al., 2024) to ~32,000(Xie et al. 2024), with most studies using several thousand images. A single biobank can be split into four non-overlapping subsets to control label leakage, or separate sources can be used for model development and GWAS(Patel et al. 2024)."*

2. GWAS of imaging traits has been a pretty long history which the introduction section does not allude to and the Phase B section mainly describes a standard GWAS pipeline and quotes tools such as BOLT-LMM and fastGWA which are standard GWAS methods (but there are many many others who can handle similar tasks). So, overall this seems to be a patched together section. Similar arguments flow for Model Interpretation section.

Overall, this section feels like an introduction to the further sections but not written as an introduction at all. I would advise the authors to restructure it to reflect more of an integrated introduction with more cohesiveness. If you want to remark about a particular method, that is okay, as long as it serves a broader purpose.

Response. Thank you for your comment. (1) History of imaging genetics. We agree the manuscript lacked historical context. We have added a new paragraph (paragraph 2) to the Introduction tracing the evolution of imaging genetics, from early candidate-gene studies (e.g., APOE ε4, 5-HTTLPR, BDNF Val66Met) through the replication crisis, to large-scale consortium efforts (ENIGMA, CHARGE), and finally to the current era of population-scale biobanks (**Line number 38-52**). (2) Restructuring the Overall Framework section. We agree that Phases B and C, as originally written, read as generic descriptions of standard GWAS and interpretation pipelines. We have revised both subsections to anchor them to what is specifically different when working with SSL-derived phenotypes. In Phase B, we open by contrasting SSL latent spaces which can comprise tens to hundreds of correlated dimensions with traditional low-dimensional IDPs, framing the need for multivariate frameworks (e.g., MOSTest, MANOVA) as a direct consequence of this high dimensionality rather than simply listing available GWAS tools. In Phase C, we explicitly contrast SSL phenotypes with expert-defined IDPs that carry built-in anatomical meaning (e.g., hippocampal volume, left ventricular mass), motivating why post hoc interpretation strategies such as decoder-based perturbation and saliency mapping are necessary for abstract latent dimensions. Also, we have expanded the "Self-Supervised Learning Approaches" section. These revisions ensure the section serves as an integrated, SSL-specific framework that motivates the organ-specific applications that follow, rather than a standalone primer on standard GWAS methodology.

3. The reference list in the SSL approaches subsection, as it often happens with reviews is never complete or comprehensive, which is understandble.

While the section provides a high-level categorization of approaches such as reconstruction, contrastive, redundancy reduction, and multimodal alignment it remains largely descriptive. But, I think these sections are missing very important references and methods, which should definitely be included.

In particular, it lacks a unifying perspective linking these methods as alternative strategies for learning invariant representations while avoiding collapse, and does not explain the underlying mechanisms that enable different approaches (e.g., negative sampling in contrastive learning or decorrelation in redundancy reduction). Important methods such as BYOL, VICReg, MoCo, and especially CLIP in the multimodal setting are absent, and there is no discussion of critical design factors like data augmentations or known limitations such as false negatives, large batch requirements, and sensitivity to data regimes. Further, the section does not connect these paradigms to downstream performance or provide even minimal mathematical explanations, which limits its depth and completeness as a review.

Response. Thank you for your comment. We have substantially rewritten the "Self-Supervised Learning Approaches" subsection (**Line number 253-384**) and added a new comparison table (**Table 2**).

1. Unifying perspective linking SSL families. We have added a framing statement that explicitly positions the reviewed SSL families as alternative strategies for learning informative, low-dimensional representations while avoiding representational collapse.The revised text then walks through how each family addresses this challenge through a distinct mechanism: reconstruction-based methods prevent collapse because a constant output cannot reconstruct the input; contrastive methods (SimCLR, MoCo) prevent it through repulsion from negative pairs; BYOL through a momentum-updated target network; and redundancy-reduction methods (Barlow Twins, VICReg) through explicit decorrelation across

latent dimensions. This collapse-avoidance framing is also summarized as a dedicated row in the new Table 2.

2. Additional methods and references. We have now added the methods flagged by the reviewer and integrated them into the appropriate subsection:
MoCo (He et al., 2020): added to the contrastive learning paragraph as the approach that decouples negative-sample count from batch size via a memory bank, addressing SimCLR's large-batch requirement.
BYOL (Grill et al., 2020): added as a negative-free contrastive variant that achieves stable representations through a momentum-updated target network.
VICReg (Bardes et al., 2022): added alongside Barlow Twins in the redundancy-reduction paragraph, with its variance-invariance-covariance decomposition described briefly.
CLIP (Radford et al., 2021): added to the multimodal alignment subsection as the foundational cross-modal contrastive framework that subsequent imaging-genetics methods (ContIG, Cross-Modal Autoencoder) adapted.
We have also noted COMICAL in the future directions section rather than in the SSL approaches subsection, because the original COMICAL study did not itself perform a GWAS and therefore falls outside our review's inclusion criteria.

3. Underlying mechanisms and design factors. The revised section now explicitly discusses the mechanisms the reviewer highlighted: the role of negative sampling in contrastive learning (and the batch-size constraint this imposes in SimCLR), decorrelation as the collapse-avoidance mechanism in Barlow Twins and VICReg, and the role of momentum target networks in BYOL. Augmentation design is discussed as a key trade-off in the new Table 2: we note that augmentation-invariance can erase heritable signal if the chosen augmentations destroy it, a GWAS-specific concern not raised in the general SSL literature.

4. Because the primary audience of this review includes geneticists and biologists, we have added a compact loss equation for each SSL family (Lrec, Lcon, Lred, Lalign), each accompanied by a plain-language interpretation in the main text, a corresponding panel in Figure 2, and a 'conceptual interpretation' row in Table 2 that restates the loss in non-mathematical terms.

5. We have added Table 2, which provides a head-to-head, GWAS-relevant comparison across the three families. Columns include the formal training objective, conceptual interpretation, collapse-avoidance mechanism, representative studies from the review, and explicit GWAS-relevant strengths and trade-offs.

4. In brain imaging genetics section, these issues remain and is a feature of the manuscript as a whole. Again some glaring omissions have to do with the lack of inclusion of methods which uses CLIP-like encoders for doing imaging-derived GWAS

a. Machado Reyes, D., Burch, M., Parida, L., & Bose, A. (2025). A foundation model for learning genetic associations from brain imaging phenotypes. Bioinformatics Advances, 5(1), vbaf196. https://academic.oup.com/bioinformaticsadvances/article/5/1/vbaf196/8233690

b. Chen, H., Liao, Y., Tang, L., Wei, X., Li, T., & Chen, W. (2025). Multivariate genome-wide analysis reveals shared genetic architecture and brain structural correlates of human cognitive abilities. Scientific Reports, 15(1), 41596.
.. and many others like this.
https://www.nature.com/articles/s41598-025-25509-z

Response. We thank the reviewer for highlighting these papers. We have carefully evaluated both suggested papers and respectfully address them as follows.

On Machado Reyes et al. (2025). COMICAL:We agree this is methodologically interesting work but believe it falls outside our review's scope. COMICAL does not derive phenotypes from images: it operates on the 154 expert-defined UK Biobank IDPs (the traditional atlas-based phenotypes our review explicitly contrasts against SSL-derived ones). It also does not perform GWAS, instead using 5,603 SNPs pre-selected from the GWAS catalog to learn many-to-many SNP–IDP associations through contrastive learning. COMICAL is more accurately characterized as a post-GWAS multimodal integration method rather than an SSL-based phenotype derivation framework. To acknowledge this related work, we have added a sentence to the Emerging Architectures section noting that CLIP-style contrastive frameworks such as COMICAL are being adapted to imaging-genetics but currently operate on pre-extracted IDPs and pre-selected variants rather than raw images.

Chen et al. (2025). This paper applies multivariate Genomic SEM to summary statistics from six cognitive trait GWAS and uses standard atlas-based IDPs only as downstream characterization via BrainXcan and Mendelian randomization. It does not involve SSL applied to raw images and would have fallen outside our scope on methodological grounds as well.

We acknowledge that our scope statement could be more explicit. Our scope is defined by two inclusion criteria: (i) the method applies SSL directly to raw or minimally-preprocessed medical images, rather than to pre-extracted expert-defined IDPs and (ii) the learned representations are used as quantitative phenotypes for genome-wide association testing.

5. There is a lack of comparison across methods and also lacking is a unifying framework which merges the reconstruction and contrastive learning-like approaches. Also missing are methodological gaps like sensitivity to multiple distributions in the cohort, bias, etc. But perhaps the most glaring omission is the lack of theoretical grounding and mathematical explanations.

Response. Thank you for your comment. The original Methods section did lack a structured comparison, a unifying lens across SSL families, and formal notation, and we agree the discussion of cohort-level methodological issues warranted strengthening. We respond to the four sub-points in turn.
1. Comparison across methods. We have added Table 2, a new cross-method comparison organized along: the representation each family encourages, the corresponding panel of Figure 2, the formal training objective, a plain-language reading of the loss, the collapse-prevention mechanism, representative studies covered in the review, and GWAS-relevant strengths and trade-offs. Figure 2 was also redrawn to mirror this taxonomy with four panels (autoencoder, contrastive, redundancy reduction, multimodal alignment), each annotated with its compact loss expression. The table separates reconstruction-based methods from the joint-embedding family, and within joint embedding distinguishes two practical sub-variants (instance discrimination on two views of the same image, versus cross-modal alignment on two modalities of the same individual). The revised text also states explicitly that "resolving which combinations work best will require head-to-head benchmarks on shared datasets with standardized pipelines," flagging the absence of a quantitative benchmark as a priority for future work.

2. Unifying framework for reconstruction-based and contrastive approaches. We have reorganized the section to make the unification visible at three levels. First, the taxonomy itself was rebuilt: what the original submission called "contrastive approaches" is now framed as one of three branches (contrastive, redundancy reduction, multimodal alignment) of a single joint-embedding family, separated from reconstruction-based methods. Second, the section now opens with an explicit shared lens: both families pursue the same underlying goal of learning informative, low-dimensional representations while avoiding representational collapse, and they differ in the mechanism by which they prevent collapse, the

reconstruction requirement itself (autoencoders), repulsion from negative pairs (SimCLR, MoCo), or explicit decorrelation across latent dimensions (Barlow Twins, VICReg). This collapse-avoidance frame runs through the entire section and is one of the comparison axes in Table 2. Third, we added a paragraph noting that the two families are not mutually exclusive and pointing to Genetic InfoMax and the cross-modal autoencoder as studies in the review that combine reconstruction and contrastive objectives.

3. Cohort heterogeneity, distributional shift, and bias.

In "Population Diversity and Generalizability" we now state explicitly that, within our literature cutoff (September 2025), every SSL imaging GWAS study identified for genome-wide discovery uses UK Biobank, a resource dominated by middle-aged participants of European ancestry, and we discuss the consequent limits on generalizability to pediatric, non-European, and under-represented populations, together with emerging non-Western biobanks (Qatar Biobank, NAKO) as candidate validation cohorts.

Second, in "Methodological and Interpretability Barriers" we now flag that SSL models may inadvertently encode non-biological variation such as scanner differences, acquisition artifacts, or ancestry-related morphological differences as latent features, potentially confounding downstream GWAS; we note that current studies mitigate this by including standard covariates (age, sex, genetic principal components) in association models, but that systematic evaluation of residual bias in learned representations remains limited.

Third, the label-leakage / circularity concern that arises when genetics enters the SSL loss and the same genotypes are subsequently tested for association with the learned embeddings is now stated explicitly in the cross-modal-alignment section and surfaced as a GWAS-relevant trade-off in Table 2.

4. Theoretical grounding and mathematical explanations. The original Methods section described SSL families in prose only, without notation or equations. We have added formal mathematical content throughout the revised section and in Figure 2. We have deliberately kept this at the level of formal definitions rather than a full theoretical treatment of SSL itself (e.g., convergence, identifiability, or sample-complexity results), which is an active research area in its own right and beyond the scope of the review, which targets biologists as readers.

6. The applications ares although discussed in some detail seem to be arbitrary.

Response. Thank you for your comment. We would like to clarify that the choice of application areas brain MRI, cardiac MRI, and retinal imaging was based on existing literature. These are the only organ systems where SSL-derived imaging phenotypes have been applied to genome-wide association studies. Our review comprehensively covers all published SSL imaging GWAS studies across these three modalities (Tables 3-5). No SSL imaging GWAS studies were identified for other organ systems (e.g., abdominal MRI, lung CT, DXA) within this timeframe, though we discuss these as promising future directions in the Challenges and Future Directions section.

7. Minor concerns:

1. Check formatting of the tables
2. Use subsection headers to delineate between sections in the applications

Response. Thank you for your comment. We have now updated the tables and have formatted section headers. We have used subsection headers to delineate between sections in the applications.

8. In summary, the manuscript addresses an important and timely topic at the intersection of self-supervised learning and imaging genetics. However, in its current form, the methodological discussion remains largely descriptive and lacks the depth expected of a review in this area. Key objectives (e.g., contrastive losses, redundancy reduction criteria, or mutual information formulations) are not explicitly defined, and there is little discussion of the principles that prevent representation bottlenecks or govern the trade-offs between competing approaches (e.g., invariance vs information preservation, interpretability vs predictive power). As a result, the presentation reads more like a catalog of methods than a critical synthesis of the field. This lack of analytical depth makes it difficult to assess when and why one class of methods should be preferred over another, particularly in the context of imaging genetics. For a review article, it is essential to go beyond surface-level descriptions and provide conceptual clarity, mathematical grounding, and critical evaluation. I strongly encourage the authors to include formal objective functions wherever appropriate, articulate underlying mechanisms and trande-offs, and synthesize literature into a coherent framework with rigor and depth. Perhaps, the authors can use an unsupervised approach to identify key manuscripts in this intersection area from PubMed and perform BERT-like topic modeling to observe different key areas and major contributions.

Response. Thank you for your comment. We have addressed the concerns in detail in our responses above. To summarize the key changes: we have rewritten the SSL Approaches subsection around a unifying collapse-avoidance framing that connects reconstruction, contrastive, redundancy-reduction, and multimodal-alignment methods as alternative strategies for learning informative low-dimensional representations; we have added compact loss expressions in the prose and in the Figure 2 caption, with a shared notation key; we have added MoCo, BYOL, VICReg, and CLIP to the main-text discussion, together with their mechanisms (negative sampling, momentum target networks, decorrelation, cross-modal contrastive alignment) and known design constraints (large-batch requirement, sensitivity to augmentation choice); we have added Table 2, a new cross-method comparison along seven GWAS-relevant axes; and we have expanded the Challenges section to discuss bias from non-biological variation encoded in latent spaces.

On the topic-modeling suggestion: we appreciate this methodological suggestion and considered it carefully. BERT-based topic modeling is well-suited to large, heterogeneous corpora where the structure of the field is not yet clear. SSL imaging GWAS literature comprises approximately ten papers across all organ systems, which is small enough that exhaustive manual review with reference tracking provides comparable or better coverage than embedding-based clustering. We did, however, use embedding-based AI search tools (Semantic Scholar, Consensus) as a complementary check during search, and we have documented our search methodology in the revised Introduction. We agree that topic modeling would become the appropriate approach as the field expands, and we have noted this as a future methodological direction. We also now include two recently published SSL GWAS studies: Chaudhary, S. et al. HistoGWAS: an AI-enabled framework for automated genetic analysis of tissue phenotypes in histology cohorts. Genome Biology 27, 122 (2026) and Zhao, X. et al. Genetic architecture of white matter microstructure captured by unsupervised deep representation learning of fractional anisotropy maps. Nature Communications 1–17 (2026).

Reviewer #2 (Remarks to the Author):

The review from Patel K and colleagues outlines methods and analytical frameworks to use imaging data as outcomes in genome wide association studies.

1. A general comment is that the text is written as a narrative review where different methodologies are described but not fully exploited and a model for integration of large datasets (images and genetic variations) is proposed. This is true for the derivation of the quantitative phenotypes from images as well as for GWAS analyses. Similar attempts have been done using different types of phenotypes and the novelty of the approach proposed does not come forward.

Response. Thank you for the comment. We would like to clarify the nature of our contribution. Since this is a narrative review, we are not proposing a new method or original analysis, but rather synthesizing an emerging body of work for a generalist audience. The novelty we highlight is not our own but that of the field: across 10 published studies, SSL-derived phenotypes have (i) identified novel genetic loci missed by traditional IDP-based GWAS (e.g., 29 novel brain loci from UDIPs, 20 novel retinal loci from OCT autoencoders, the STRN–mitral valve orientation locus in cardiac imaging), (ii) yielded higher heritability than expert-defined IDPs in several studies (eg $h^2 \approx 0.25$ for UDIPs (vs. $h^2 \approx 0.18$ for the expert-defined brain IDP comparator reported in the same study), and $h^2 \approx 0.38$ for Genetic InfoMax), and (iii) scaled to new imaging modalities without requiring modality-specific segmentation pipelines. We have now strengthened this framing in the Introduction and added Table 1 to make the complementary value of SSL vs. expert-defined IDPs explicit. We have included a forward-looking roadmap in Box 1 outlining the components an integrated SSL imaging-genetics framework would require.

2. The tables are impossible to read, they lack references and a clear context. Do they refer to methods the Authors or other have developed?

Response. Thank you for your comment.

Authorship of the methods. The tables summarize methods and findings from published studies by multiple independent research groups. We have clarified this in the table captions, which now explicitly state that each row corresponds to a primary study by the cited authors.

References. Each study entry now includes its corresponding citation, so readers can immediately identify the source.

Readability. We have reformatted the tables: removed dense technical columns (Preprocessing, Latent Space, Mean h^2, Interpretability), consolidated the cohort-size column, added study citations to each row, and unified column structure across all three organ tables (Tables 3-5). The simplified tables now retain only the columns most relevant for a generalist comparison.

3. In which way reconstruction of images, and the genetic analyses of it should translate in progress?

Response. Thank you for your comment. We have now clarified this point in the revised manuscript in the revised Self-Supervised Learning Approaches section *"Reconstruction-based methods learn representations by compressing an input through a low-dimensional bottleneck and then reconstructing it (Figure 2A). To succeed under this constraint, the model must filter out noise and redundancy and retain the most salient structural and semantic information. The resulting bottleneck representation, not the reconstruction itself, is what is used as a GWAS phenotype. A classic autoencoder maps an input x to a latent vector* $z = f_\theta(x)$ *and decodes it back to* $\hat{x} = g_\phi(z)$*, training to minimize reconstruction error:* $L_{rec} = \| x - \hat{x} \|^2$*. In plain terms, the model is penalized whenever the reconstructed image differs*

from the original (Figure 2A), so the encoder is forced to pack the most informative anatomical content into the latent bottleneck[37,64].”

The goal of reconstruction-based SSL is not the reconstructed image but the compressed latent representation learned in the process passing high-dimensional imaging data through a low-dimensional bottleneck retains the most informative structural features, which are then used as quantitative phenotypes for GWAS. Concrete progress from this approach includes novel locus discovery (29 novel brain loci from UDIPs; 20 novel retinal loci from OCT autoencoders) and improved downstream risk stratification for glaucoma and cardiovascular disease.

4. We use already measures that are derived from images, as an example calcium score for intimal calcium, but we use it because it represent a specific stage of the disease. In which way the transformations described can be applied to improve our understanding of a disease/process?

Response. Thank you for your comment. Traditional image-derived measures like coronary calcium score have direct clinical interpretability as they correspond to a known stage of disease. SSL-derived latent features, by contrast, are abstract and do not carry built-in biological meaning. However, this abstraction is also their potential strength. SSL features can capture distributed morphological patterns that do not correspond to any single predefined measurement but are nonetheless heritable and biologically meaningful. For example, the STRN locus identified through SSL-derived cardiac phenotypes was associated with a change in mitral valve orientation without altering LV volume a geometric relationship that standard volume-based IDPs would not detect (Bonazzola et al., 2024).

That said, we acknowledge that translating SSL-derived features into mechanistic disease understanding remains an open challenge. Unlike established biomarkers with decades of clinical validation, SSL imaging genetics is an emerging field and no community consensus yet exists on how to systematically link abstract latent dimensions to specific disease processes. We have added this as a limitation in the Challenges and Future Directions section. We have also added Table 1, which compares expert-defined IDPs and SSL-derived phenotypes across definition, interpretability, scalability to new modalities, phenotypic coverage, reproducibility, processing speed, and best-suited applications, and we discuss these complementary strengths in the revised Introduction.

Reviewer #3 (Remarks to the Author):

I co-reviewed this manuscript with one of the reviewers who provided the listed reports. This is part of the Communications Biology initiative to facilitate training in peer review and to provide appropriate recognition for Early Career Researchers who co-review manuscripts. Thanks for the editor’s invitation.
In this review, the authors aim to argue that self-supervised learning (SSL) can be used to derive data-driven imaging phenotypes from raw medical images, and that these latent traits can then be used in GWAS to identify potential signals beyond traditional expert-defined image-derived phenotypes. Overall, the authors present SSL-based imaging genetics as a promising strategy for biobank-scale discovery, especially in settings where conventional IDPs may miss subtle and high-dimensional anatomical variation.

1. As we all work in the field of AI + medicine, to some extent, I agree with the authors that machine learning, or more broadly AI algorithms, may facilitate better extraction of imaging signals and thereby enrich genetic discovery. However, I feel that the review is currently too method-enumerative. The manuscript spends substantial space listing architectures, objectives, preprocessing steps, and pipelines, but offers less conceptual synthesis. As written, it reads more like a catalogue of methods than a critical review explaining what these approaches have collectively taught us biologically, and under what circumstances one strategy may be preferable to another.

Response. Thank you for this summary critique. We have made several manuscript-wide changes to simplify the presentation and foreground a central message. We have added Table 1, which provides a systematic comparison of expert-defined IDPs and SSL-derived phenotypes across definition, interpretability, scalability to new modalities, phenotypic coverage, reproducibility, processing speed, and best-suited applications. We have added Box 1 summarizing current challenges and future directions, and expanded the Challenges section to address limited external validation, overfitting to UK Biobank, the risk that SSL models may encode non-biological variation, and the absence of experimental follow-up of SSL-GWAS loci. We have streamlined Tables 3-5 (the organ-specific summary tables for brain, cardiac, and retinal imaging) by removing technical columns and adding study references, making them more accessible to a generalist audience. The SSL Approaches subsection now connects all method families as alternative strategies for avoiding representational collapse, and notes that reconstruction and contrastive objectives are not mutually exclusive. Finally, Table 2 compares major SSL approaches and clarifies the contexts in which each strategy may be preferable.

2. Another issue is the scope of the review itself. Although the title emphasises self-supervised imaging phenotypes, the manuscript appears to use "SSL" rather broadly, including both selfsupervised and unsupervised models. This blurs the conceptual boundary of the field being reviewed. I think this point should be clarified more carefully, or alternatively, the title should be broadened to better reflect the actual content.

Response. Thank you for your comment. We added clarification in the introduction.
"Although SSL is a subclass of unsupervised learning, SSL methods rely on supervised loss functions which distinguish them from traditional unsupervised learning such as cluster, dimension reduction, and density estimation. Although autoencoders were invented before the term SSL got popular, they are often categorized as an SSL because that the idea of deriving a compact representation (bottleneck) and using the decoder as a guide to train the encoder are very much in line with the philosophy of SSL."

3. A further concern is the biological interpretation of these latent features. When features are extracted from machine learning or neural network models, how can these hidden representations be understood by clinicians or biomedical researchers? The review strongly emphasises discovery yield and technical performance, but gives less critical discussion of what these latent phenotypes actually represent biologically, and whether they truly function as meaningful intermediate traits rather than simply high-performing embeddings. This point is especially important in medical research. In contrast to ImageNet-like classification tasks (which may not be a perfect example, as they are supervised learning tasks), where strong predictive performance may already be considered convincing, medical applications usually require more than predictive accuracy alone, and so do these hidden features. We need to understand whether the learned features reflect meaningful, reproducible, and biologically grounded patterns before they can be confidently interpreted or translated. I may be wrong here, and this can of course be discussed, especially because nowadays ChatGPT and other transformer-based models seem to overlook interpretation to some extent while still achieving very strong performance.

Response. Thank you for your comment. It is important to know whether SSL-derived phenotypes function as meaningful intermediate traits or simply as high-performing embeddings. We agree this distinction matters more in medical genetics than in many other ML contexts, because the unit of downstream inference is a genetic locus and its biological consequence, not a prediction score. A locus discovered against an uninterpretable phenotype is harder to act on than one discovered against a phenotype with biological meaning, regardless of statistical significance. Three lines of evidence in the reviewed studies bear on whether SSL embeddings reflect biology rather than scanner or population artifacts. First, SNP-heritability: heritable embeddings are more likely to reflect biological variation than noise or technical confounders, and the reviewed studies report substantial heritabilities: UDIPs $h^2 \approx 0.25$, exceeding the expert-defined brain IDP comparator ($h^2 \approx 0.18$) reported in the same study (Patel et al., 2024); Genetic InfoMax $h^2 \approx 0.38$ (Xie et al., 2023). Second, convergence with known biology: the cardiac cross-modal autoencoder analysis recovered well-established cardiovascular genes (NOS1AP, TTN, SCN10A, SLC35F1; Radhakrishnan et al., 2023), providing external grounding that the embeddings reflect known cardiac biology rather than purely statistical artifacts. Third, post hoc anatomical attribution via encoder- or decoder-based methods: perturbation-based decoding linking latent dimensions to ventricle and thalamus structure in UDIPs (Patel et al., 2024), visual shape-effect analysis linking the STRN locus to mitral valve orientation in cardiac graph-autoencoder GWAS (Bonazzola et al., 2024), and thickness difference maps linking SSL dimensions to paracentral retinal thinning in diabetes (Sergouniotis et al., 2024). These illustrate that abstract latent dimensions can be mapped back to specific anatomical structures.

We note in the manuscript that "perturbation of a single latent dimension can propagate globally in the decoded image, making anatomical attribution less precise than voxel-wise IDPs," and that "saliency maps can saturate, obscuring the magnitude of specific features." Critically, no SSL imaging GWAS study within our cutoff has provided experimental follow-up (functional genomics, animal models) that would close this gap, and we now state this explicitly in Box 1 and in the Methodological and Interpretability Barriers section.

Phase C of the framework section covers the main encoder- and decoder-based interpretation strategies; each organ-specific section then discusses how individual studies applied them; and Table 1 highlights interpretability as a key axis distinguishing SSL from expert-defined approaches.

4. In addition, many of the reviewed studies rely heavily on very large biobank datasets and focus mainly on discovery. The review would be stronger if it more directly addressed issues such as limited external validation, generalizability across cohorts, and the interpretability of learned features. Even within UK Biobank-based studies, one may still question whether some current medical AI applications are too optimized for a specific dataset and publication setting, rather than demonstrating true robustness. The lack of strong validation datasets, and the fact that some patterns may only appear in a few unusually large biobanks, raise real concerns about generalizability. Although the manuscript does acknowledge some of these challenges, I feel that the critique remains relatively soft, and the limited discussion of biological interpretation makes this issue even more problematic.

Response. Thank you for your comment. Our manuscript updates now addresses these limitations directly.

1. Limited external validation**.** All our tables show external validation as a field. No study has demonstrated replication in a non-UK Biobank dataset. We acknowledge this as a significant limitation in the challenges and future direction section.
2. Overfitting to UK Biobank. We have added a more direct discussion of the concern that SSL models may be optimized for UK Biobank-specific characteristics, its uniform scanner (1.5T Siemens for cardiac, 3T Siemens for brain), standardized acquisition protocols, and predominantly European-ancestry, middle-aged population.
   "*A related concern is that SSL models may inadvertently encode non-biological variation such as scanner differences, acquisition artifacts, or ancestry-related morphological differences as latent features, potentially confounding downstream GWAS. Current studies mitigate this by including standard covariates (e.g., age, sex, genetic principal components) in association models, but systematic evaluation of residual bias in learned representations remains limited.***"**
3. We have strengthened the critique that current interpretability methods remain largely post hoc and qualitative. "*Interpretability remains a persistent difficulty. While perturbation-based decoding and saliency mapping provide partial insights, they have concrete limitations: decoder perturbations often smear effects across the anatomical boundaries rather than localized, and saliency maps can saturate, obscuring the magnitude of specific features. Clinical translation from SSL-discovered loci to therapeutic targets also remains largely theoretical, with experimental follow-up of SSL-GWAS loci essentially absent from the literature.*"
4. We added the following cautionary statement to the Abstract: "Early studies suggest that these data-driven approaches can complement traditional IDPs and may expand the phenotypic space available for discovery of heritable imaging traits, although broader validation across cohorts, ancestries, scanners, and imaging protocols remains needed."
5. At the end of the Introduction, we added: "While challenges remain in reproducibility, cross-cohort generalization, interpretability, and biological validation, SSL-derived phenotypes represent a promising complement to expert-defined IDPs and may scale to biobank-level analyses; however, their broader adoption will require standardized benchmarking, external replication, and validation across diverse biobank and clinical cohorts."
6. Statements in each section showing the limitations of each approaches.

5. Overall, this is a timely and potentially interesting review, but in its current form it feels somewhat descriptive and method-driven. I think the manuscript would benefit from a clearer scope, stronger conceptual synthesis, and more critical discussion of biological meaning, interpretability, and generalizabilit

Response. Thank you for your comment. We summarize the revisions below and articulate the central message of the review.
Central message. SSL-derived imaging phenotypes represent a methodologically distinct approach to imaging genetics: rather than relying on predefined anatomical measurements, the imaging data itself defines what features are most informative for downstream genetic analysis. Across the approximately 11 published studies in brain, cardiac, and retinal imaging within our cutoff, this approach has yielded novel loci missed by traditional IDP-based GWAS, higher SNP-heritability than matched expert-defined comparators in several studies, and scalability to imaging modalities without modality-specific annotation pipelines. At the same time, SSL phenotypes are abstract, their biological meaning must be inferred post hoc, and the field remains almost entirely dependent on a single cohort (UK Biobank). Our review synthesizes these trade-offs to help researchers evaluate when and how SSL approaches can complement traditional methods.
Specific revisions in response to this comment.

1. Clearer scope. The Introduction now states explicitly that this is a narrative review providing a methodological and biological framework for SSL imaging genetics, with two formal inclusion criteria: (i) SSL applied directly to raw or minimally-preprocessed medical images, and (ii) the learned representations used as quantitative phenotypes for GWAS. We have also documented our search strategy (databases, reference tracking).
2. Stronger conceptual synthesis: unifying SSL framework. We have rewritten the SSL Approaches section around a unifying collapse-avoidance lens that connects reconstruction-based and joint-embedding methods as alternative strategies for learning informative low-dimensional representations while preventing representational collapse. Within joint embedding, contrastive (SimCLR, MoCo, BYOL), redundancy-reduction (Barlow Twins, VICReg), and multimodal-alignment (ContIG, CLIP-style) methods are presented as three branches of a single family, each defined by how it avoids collapse. We have added Table 2, which compares these families along seven GWAS-relevant axes (representation goal, training objective, collapse-prevention mechanism, representative studies, GWAS strengths, GWAS trade-offs).
3. Biological meaning and interpretability. We have added a systematic comparison of expert-defined IDPs and SSL-derived phenotypes (Table 1), making the distinction between built-in vs. post hoc interpretability explicit. We provide concrete examples of biology revealed by SSL that traditional IDP approaches do not capture (e.g., mitral valve orientation changes at the STRN locus; Bonazzola et al., 2024), and we acknowledge that systematic linkage of abstract latent dimensions to specific disease processes remains an open challenge.
4. Generalizability. We have expanded the Population Diversity and Generalizability and the Methodological and Interpretability Barriers subsections to cover ancestry imbalance (every SSL imaging GWAS to date uses UK Biobank), domain shift between pretraining and downstream cohorts, the risk that SSL models encode scanner/site/acquisition artifacts as latent features, and the label-leakage concern when genetics enters the SSL loss.

Reviewer #4 (Remarks to the Author):

This manuscript provides a clear and well-structured overview of recent developments in self-supervised learning approaches for imaging genetics. The review is informative and scientific sound.
However, a few minor issues could be addressed to improve the the manuscript.

1. The manuscript states that a literature search was conducted, but the criteria used to select the studies included in the review are not described. Providing a brief description of the search strategy (e.g., databases consulted, keywords, or general inclusion criteria) would improve transparency and help readers better understand how the reviewed studies were identified.

Response. Thank you for your comment. We would like to clarify that this is a narrative review rather than a systematic review, and as such does not follow a formal systematic search protocol (e.g., PRISMA). However, we agree that transparency in how studies were identified is important. We have expanded the scope paragraph in the Introduction to describe our search strategy.

"*Our scope is defined by two inclusion criteria: (i) the method applies SSL directly to raw or minimally-preprocessed medical images, rather than to pre-extracted expert-defined IDPs and (ii) the learned representations are used as quantitative phenotypes for genome-wide association testing. This review aims to provide a methodological and biological framework for understanding how SSL-derived imaging phenotypes support genetic discovery. Our literature search covered papers, identified through database searches (PubMed, Google Scholar) and reference tracking of included studies.*"

2. Some statements comparing performance across studies appear somewhat definitive. For example, the manuscript notes that Genetic InfoMax "achieved the highest heritability among current approaches." Given that the reviewed studies differ in preprocessing pipelines, phenotype definitions, and GWAS sample sizes—as also acknowledged later in the text—such cross-study comparisons may be difficult to interpret directly. It may therefore be preferable to use slightly more cautious wording (e.g., "reported higher heritability" or "among the highest reported") to avoid implying a direct methodological ranking.

Response. Thank you for your comment. We have now removed the statement about heritability in the text and in all the tables as such cross-study comparisons may be difficult.
"Genetic InfoMax reported heritability of 0.38~~achieved the highest heritability among current approaches ($h^2 \approx 0.38$)~~ and improved genetic discovery with 40 loci (15 unique to this method) for the top 10 principal components (PCs) of the endophenotypes. However, unlike UDIPs, GIM did not include a dedicated interpretability pipeline or replication cohort, and the choice of using top PCs as phenotype was meant for methodological development rather than maximizing biological discovery. Therefore, further rigorous studies at scale are warranted." We added the following cautionary statement to the Abstract: "Early studies suggest that these data-driven approaches can complement traditional IDPs and may expand the phenotypic space available for discovery of heritable imaging traits, although broader validation across cohorts, ancestries, scanners, and imaging protocols remains needed."
At the end of the Introduction, we added: "While challenges remain in reproducibility, cross-cohort generalization, interpretability, and biological validation, SSL-derived phenotypes represent a promising complement to expert-defined IDPs and may scale to biobank-level analyses; however, their broader adoption will require standardized benchmarking, external replication, and validation across diverse biobank and clinical cohorts."

Reviewer #5 (Remarks to the Author):

The authors provide a review of self-supervised learning (SSL) approaches for extracting AI-derived endophenotypes from medical imaging data. The manuscript introduces the general framework of SSL methods, followed by discussions on downstream GWAS and model interpretation strategies. In addition, the authors summarize several organ-specific applications of SSL in imaging genetics, including studies of the brain, heart, and retina, and highlight key findings regarding model architectures and genetic discoveries. Finally, the review outlines current challenges and future directions in SSL-based imaging genetics, such as limited dataset availability, European ancestry bias, and methodological and interpretability barriers. Overall, this manuscript provides a useful overview of the emerging field of SSL-based imaging genetics. However, before publication, I have some comments and suggestions that may help improve the clarity and comprehensiveness of the review.

1. In the Introduction, the authors present a rather critical perspective on expert-defined imaging-derived phenotypes (IDPs) while emphasizing the advantages of SSL-derived phenotypes. While it is true that expert-defined IDPs have limitations, such as sensitivity to image quality and reliance on existing anatomical knowledge, they have also played a crucial role in many successful imaging-genetics studies. It would therefore be helpful if the authors could provide a more systematic comparison between expert-defined IDPs and SSL-derived phenotypes. For example, discussing their respective strengths, limitations, and suitable application scenarios would help readers better understand the practical advantages of SSL approaches.

Response. Thank you for your comment. We have revised the Introduction to frame SSL-derived phenotypes as complementary to, rather than a replacement for, expert-defined IDPs. The revised text now reads: "*Expert-defined IDPs and SSL-derived phenotypes offer complementary strengths: IDPs provide direct clinical interpretability, reproducibility across processing pipelines, and established biological meaning, whereas SSL approaches can capture broader and more distributed variation beyond predefined measurements and scale more readily to new imaging modalities, although interpretability and biological grounding remain challenges.*"
With your feedback, we have now added **Table 1** providing a systematic comparison of expert-defined IDPs and SSL-derived phenotypes across key dimensions including interpretability, scalability, reproducibility, phenotypic coverage, and suitable application scenarios.

2. In the second paragraph of the Introduction and in the Discussion section, the authors briefly mention several imaging cohorts and biobanks other than the UKB, and the review primarily focuses on studies based on UKB data. Although these additional datasets are generally smaller in scale, it would still be valuable for the authors to summarize the main findings from studies using these cohorts. Providing a brief overview of their characteristics, strengths, and limitations could offer useful insights and potentially guide future study designs for other cohorts and emerging imaging-genetics projects.

Response. Thank you for your comment. We have now mentioned in the Population Diversity and Generalizability that *"To the best of our knowledge, every SSL imaging GWAS study identified for genome-wide discovery uses UK Biobank"* . To stay within the scope of our review, which focuses specifically on SSL-derived phenotypes for GWAS, we describe findings from these SSL studies rather than summarizing the broader traditional imaging GWAS literature from other cohorts. We discuss how emerging resources such as NAKO and the Qatar Biobank could enable the first non-UKB SSL imaging GWAS and critically needed cross-cohort replication in the Challenges and Future Directions section.

3. In Lines 130–133, the authors mention that PCA can be used as an efficient dimensionality reduction method when the number of latent features is large. While PCA is indeed widely used, it would be beneficial to discuss additional strategies for summarizing or integrating latent representations, as this step is crucial for downstream analyses and can also help identify biologically meaningful patterns. For example, Yang, Z., Wen, J., Erus, G. et al. Brain aging patterns in a large and diverse cohort of 49,482 individuals. Nat Med 30, 3015–3026 (2024). They proposed R-indices to summarize brain aging patterns across multiple anatomical regions. https://pmc.ncbi.nlm.nih.gov/articles/PMC11483219/

Response. Thank you for your comment. We have added a brief mention of alternative dimensionality reduction strategies beyond PCA, including the R-indices framework (Yang et al., 2024), which learns a small set of indices where each corresponds to a specific pattern of anatomical change. *"Other approaches, such as R-indices, learn a small set of indices, with each index capturing a distinct pattern of anatomical variation rather than maximizing statistical variance, as PCA does[47]."* We cite this work as a methodological alternative for the dimensionality-reduction step rather than as one of the studies reviewed: it operates on pre-extracted ROI volumes rather than raw images and does not perform GWAS on the learned indices, placing it outside our two inclusion criteria. We agree it illustrates how structured latent representations can complement PCA for deriving more interpretable phenotypes from SSL embeddings, and we have framed the citation accordingly in the revised text.

4. The authors discuss SSL applications in three major organ systems (brain, cardiac, and retinal imaging), but I believe the discussion is not comprehensive enough. For these three organ-specific applications, there appears to be only 2-3 pieces of relevant evidence for each organ. In addition, the manuscript briefly mentions other organ systems (e.g., skeletal imaging), but these are not discussed in detail. I am aware that the available literature is limited, the authors could include a short paragraph summarizing emerging applications in other organ systems.

Response. Thank you for your comment. We acknowledge that the number of studies per organ system is small (2-4 per modality). This reflects the current state of the field: SSL imaging GWAS is an emerging area with approximately 10 published studies across all organ systems. Our review comprehensively covers all studies meeting our inclusion criteria (Tables 2-5). We have added the recently published UDIP-FA study to the Brain Imaging Genetics section and Table 3. We have also revised the Future Directions section to discuss additional underexplored modalities, including abdominal MRI, DXA, lung CT, and histopathology. For histopathology, we now cite HistoGWAS as an emerging example showing that pretrained histology encoders can derive quantitative tissue embeddings for GWAS.

"Beyond architectural advances, extending SSL imaging genetics to currently underexplored modalities such as abdominal MRI, DXA, lung CT, and histopathology where supervised methods have already demonstrated substantial heritable signal represents a near-term opportunity to expand the scope of genetic discovery. For example, HistoGWAS recently demonstrated that pretrained histology encoders can be used to derive quantitative tissue embeddings for GWAS, showing histopathology as an emerging direction for image-based GWAS beyond conventional radiological imaging."

5. Given the complexity of SSL models, the review could also address issues related to reproducibility, including the availability of pretrained models, standardized preprocessing pipelines, and open benchmarking datasets for imaging-genetics studies. These aspects may represent important future directions for the development and broader adoption of SSL-based approaches.

Response. Thank you for your comment. Our Challenges and Future Directions section now include an updated Benchmarking and Standardization paragraph and now we have made

relevant changes to address these concerns. *"Benchmarking and Standardization. To usher the field into maturation, we need the development of standardized benchmarks and open evaluation frameworks, which would allow consistent comparison of SSL models across datasets and ensure reproducibility. These benchmarks should explicitly track metrics such as heritability per latent dimension, average replication p-values for top hits, and compute/energy costs to allow for transparent comparison of SSL architectures. Equally important is the release of pretrained models and standardized preprocessing pipelines, which would lower barriers to entry for research groups without extensive computational resources and enable direct comparison of downstream GWAS results across studies. Recent work has emphasized systematic benchmarks; for example, EmbedGEM evaluates whether embeddings are both genetically informative and clinically meaningful(Mukherjee et al. 2024)."*

References:

Agrawal, Saaket, Minxian Wang, Marcus D. R. Klarqvist, et al. 2022. "Inherited Basis of Visceral, Abdominal Subcutaneous and Gluteofemoral Fat Depots." *Nature Communications* 13 (1): 3771.

Baird, Denis A., Daniel S. Evans, Frederick K. Kamanu, et al. 2019. "Identification of Novel Loci Associated With Hip Shape: A Meta-Analysis of Genomewide Association Studies." *Journal of Bone and Mineral Research : The Official Journal of the American Society for Bone and Mineral Research* 34 (2): 241–251.

Elliott, Lloyd T., Kevin Sharp, Fidel Alfaro-Almagro, et al. 2018. "Genome-Wide Association Studies of Brain Imaging Phenotypes in UK Biobank." *Nature* 562 (7726): 210–216.

Gui, Jie, Tuo Chen, Jing Zhang, et al. 2024. "A Survey on Self-Supervised Learning: Algorithms, Applications, and Future Trends." *IEEE Transactions on Pattern Analysis and Machine Intelligence* 46 (12): 9052–9071.

Liu, Xiao, Fanjin Zhang, Zhenyu Hou, et al. 2020. "Self-Supervised Learning: Generative or Contrastive." In *arXiv [cs.LG]*. June 15. arXiv. https://doi.org/10.48550/arXiv.2006.08218.

Liu, Yi, Nicolas Basty, Brandon Whitcher, et al. 2021. "Genetic Architecture of 11 Organ Traits Derived from Abdominal MRI Using Deep Learning." *eLife* 10 (June). https://doi.org/10.7554/eLife.65554.

Manichaikul, Ani, Eric A. Hoffman, Joanna Smolonska, et al. 2014. "Genome-Wide Study of Percent Emphysema on Computed Tomography in the General Population. The Multi-Ethnic Study of Atherosclerosis Lung/SNP Health Association Resource Study." *American Journal of Respiratory and Critical Care Medicine* 189 (4): 408–418.

Molen, Marieke C. van der, Jorine E. Hartman, Cornelis J. Vermeulen, et al. 2021. "Determinants of Lung Fissure Completeness." *American Journal of Respiratory and Critical Care Medicine* 204 (7): 807–816.

Tobias, Jonathan H., Maria Nethander, Benjamin G. Faber, et al. 2024. "Femoral Neck Width Genetic Risk Score Is a Novel Independent Risk Factor for Hip Fractures." *Journal of Bone and Mineral Research : The Official Journal of the American Society for Bone and Mineral Research* 39 (3): 241–251.

Yang, Zhijian, Junhao Wen, Guray Erus, et al. 2024. “Brain Aging Patterns in a Large and Diverse Cohort of 49,482 Individuals.” *Nature Medicine* 30 (10): 3015–3026.

Dear Dr. Inglis,

Thank you for the opportunity to revise our manuscript and for your helpful feedback. We have addressed all the comments below and have attached the revised manuscript and the manuscript with marked changes. We also attach the editorial checklist.

Editor Comments

Dear Dr. Zhi,

Thank you again for your patience and for submitting your revised manuscript "A Review of Self-Supervised Imaging Phenotypes for Genome-Wide Association Studies" to Communications Biology. We have now received reports from 4 reviewers and, based on their comments, we have decided to invite a revision of your work. You will find the reviewers' reports below. As you can see, though they found your work to be improved and of continued interest, they raised important points which must be addressed in your revised paper. We would generally ask that you address the remaining discussion points from Reviewers #1 and #5, including the potential of a "decision tree" figure to provide advice to readers (per Reviewer #1). As before, we are happy to discuss the revisions in more detail, or look over a draft of the manuscript prior to resubmission.

Please edit your revised manuscript according to requests listed in the online table, here: here.

Please **read and complete the table in the link**, as we will be unable to further assess your revised manuscript until these editorial requirements are addressed, and your manuscript will be returned to you for further action.

Please outline all revisions made in the right-hand column of the table and include any relevant notes that may aid us in assessing your revised work.

When resubmitting, please include:

- A point-by-point response to the reviewers' comments. If you are unable to address specific reviewer requests or find any points invalid, please explain
- A clean version of your revised manuscript with no mark-ups
- A marked-up version of your paper with all changes highlighted in a different colour

Thank you for your valuable comments. We have completed the editorial checklist (online Word document) and confirmed that we have met all editorial requirements. We have included a new Table 3, "Decision map for selecting self-supervised learning approaches for imaging-phenotype GWAS" as a response to reviewer comments. To ensure we don't exceed 8 display items, we have combined the section-specific tables (brain, cardiac, and

retina) into one common table, “Table 4. Summary of self-supervised learning (SSL) approaches across brain, cardiac, and retinal imaging genetics.”

Referee #1: ML, GWAS

Referee #2: Cardiovascular imaging, genetics

Referee #4: ML, genomics, brain disease

Referee #5: ML, GWAS, IDPs

Reviewers' comments:

Reviewer #1 (Remarks to the Author):

I thank the authors for revising the manuscript substantially. However, several of my previous concerns remain along with some fresh concerns.

1. With the inclusion of the clear statement of the inclusion criteria, I think the motivation to study SSL directly applide to raw or minimally-preprocessed images and learned representation based GWAS is a bit too narrow of a focus. On top of this, I also think it is under-motivated. I think for a review in Comms Bio, the introduction should clearly establish the biological problem and give necessary motivation for the problem and then argue that SSL on images along with GWAS is a route that is often taken and hence needed to be reviewed.

The authors should distinguish more clearly between the topics they discuss: (a) SSL as a representation learning objective (b) medical image phenotyping, and (c) GWAS on image-derived traits. The present introduction quickly arrives at the inclusion criteria before laying out the biological motivation.

Thank you for the comment. We have revised the introduction to describe the biological challenge of deriving scalable, heritable imaging traits that preserve meaningful variation. We now more explicitly distinguish between SSL as the representation-learning objective, SSL-based medical-image phenotyping as the derivation of quantitative imaging traits, and GWAS as the downstream genetic-association analysis. The inclusion criteria are presented only after establishing this biological and conceptual motivation.

Manuscript changes: Miscellaneous changes in introduction.

2. I think the authors should refrain from statements like "SSL methods rely on supervised loss functions" as this is not precise. SSL often uses supervised-style optimization objectives or pseudo-label tasks but not explicit phenotype/trait labels. These nuances should be mentioned and phrased carefully.

Thank you for this clarification. We agree that describing SSL as relying on “supervised loss functions” was imprecise. We have revised the text to distinguish the source of supervision from the optimization objective. SSL may use supervised-style predictive losses or pseudo-label tasks, but the training targets are derived from the data itself rather than from externally provided phenotype or trait labels.

Manuscript changes:

Although SSL is generally considered a form of unsupervised representation learning, it uses explicit predictive objectives whose targets are generated from the input data or naturally paired views, rather than from externally provided phenotype or trait labels. This distinguishes SSL from conventional unsupervised approaches such as clustering, dimensionality reduction, and density estimation.

3. Balance the Table 1 in more realistic/practical terms. The table frames expert-defined IDP as too rigid and limited. Many SSL algorithms still use preprocessing choices, organ/ROI selection, etc. architecture designs which learns with some form of anatomical prior. So, saying "SSL-derived phenotypes learn without anatomical prior" is too strong. The table should also acknowledge that SSL embeddings can encode non-biological variation such as batch effects, demographic structure, or image artifacts. As this review targets genetic discovery, the table should explicitly compare the two phenotype classes in terms of post-GWAS interpretability, burden tests, and biological validation.

Thank you for this important suggestion. We agree that the previous version of Table 1 presented expert-defined IDPs and SSL-derived phenotypes too categorically. We have revised the table to emphasize that both approaches depend on image acquisition, preprocessing, quality control, technical choices, and cohort characteristics. We removed the statement that SSL-derived phenotypes are learned “without anatomical priors” and now clarify that anatomical assumptions may enter through registration, organ or region-of-interest selection, augmentation, input construction, and architecture design. We also acknowledge that SSL embeddings may encode non-biological variation, including scanner and batch effects, demographic structure, and imaging artifacts. Finally, we expanded Table 1 to compare the two phenotype classes in terms of GWAS testing burden and post-GWAS interpretability.

Manuscript changes: Miscellaneous changes in Table 1.

4. The field of SSL-based brain imaging/cardiac/retinal genetics is emerging, but the evidence base summarized here remains small and highly heterogeneous. The manuscript needs to articulate why this nascent field requires a standalone review rather than a section within a broader review of imaging genetics, SSL-derived medical-image phenotypes, or deep-learning-based GWAS phenotyping. As written, the motivation rests heavily on a small set of studies, while the synthesis does not yet extract sufficiently general methodological or biological lessons from them.

Thank you for your valuable comment. We would like to provide the motivation for the focused review, which lies not in the number of published studies but in the distinctive methodological problems created when learned image representations are converted into quantitative phenotypes for GWAS. These include phenotype construction, dimensionality and multiple-testing burden, separation of model-training and GWAS samples, circularity in genotype-informed learning, heritability assessment, replication, and post-GWAS interpretation. Broader reviews of imaging genetics (Bian et al.) or medical-image SSL (Krishnan et al.) do not usually address this full workflow.

Bian, Y., Akey, J.M. Genetic analysis of imaging-derived phenotypes. Nat Rev Genet (2026). https://doi.org/10.1038/s41576-026-00989-5

Krishnan, R., Rajpurkar, P. & Topol, E. J. Self-supervised learning in medicine and healthcare. Nat. Biomed. Eng. 6, 1346–1352 (2022).

5. The tables indicating how many loci were found is informative but it would be good to see if the learned phenotypes are reproducible, nonredundant (compared with established IDPs), biologically interpretable, clinically relevant (variant effects can be studied), etc.

Thank you for this helpful suggestion. Our original organ-specific tables included detailed information on interpretability and related study characteristics. However, these tables had become too detailed and difficult to navigate, so some columns were removed in the previous revision based on suggestions from the editor and other reviewers. To improve clarity and comply with the journal’s limit on display items, we have now consolidated the organ-specific tables into a single cross-organ summary table and simplified its presentation. The published literature remains limited and heterogeneous, and many studies do not systematically evaluate phenotype reproducibility, non-redundancy relative to established IDPs, clinical relevance, or variant-level interpretation. We therefore summarize these features in the main text wherever evidence is available.

6. There are numerous issues with the language (copy editing), missing commas, the tables are not well-wrapped and difficult to follow. The organization of the tables need clear improvement.

Thank you for highlighting these presentation issues. We have thoroughly reviewed the manuscript to resolve the issues and have improved the organization and readability of the tables.

Manuscript changes. Miscellaneous changes throughout the manuscript.

7. I think it would greatly benefit the field if the authors include a decision map as a main takeaway of their review. This table can serve as a summarized, one-stop place to allow people with relevant biological tasks decide on which SSL methods to choose, what their (dis)advantages are, etc.

Thank you for your valuable suggestion. We have now added table 3 which should serve as a decision map. We consolidated our imaging modality specific tables into one table.

Manuscript changes.

Table 3. Decision map for selecting self-supervised learning approaches for imaging-phenotype GWAS

Goal	SSL Approach	Representative implementation	Rationale	Limitations
**Hypothesis-free discovery across a whole organ; no strong prior on region**	Reconstruction: e.g., 3D convolutional AE	UDIPs for brain MRI[37], autoencoder model for OCT images[40]	The bottleneck retains distributed anatomical variation, and the decoder supports post hoc attribution.	Latent dimensions may be correlated; the bottleneck may also retain scanner and acquisition variation.
**Mapping regional anatomy is important**	Reconstruction with an explicit decoder (AE or VAE)	UDIPs for brain MRI[37]	Latent vectors can be perturbed and decoded back into image space.	Localization is less precise than voxel-wise IDPs.
**Input is a surface, mesh, or shape rather than a voxel grid**	Geometric learning: Graph-convolutional AE	Unsupervised Phenotype Ensemble model for left ventricular meshes for cardiac MRI[46]	Preserves mesh topology; locus-associated shape effects can be visualized across latent quantiles.	Depends on the quality of upstream segmentation and registration.
**Many latent dimensions will be tested individually, and redundancy among phenotypes should be minimized**	Redundancy reduction: Barlow Twins or VICReg	Champollion V0 model for anterior cingulate sulci[62]	Training objectives discourage cross-dimension redundancy, reducing repeated representation of similar information across latent phenotypes.	No decoder; anatomy must be attributed indirectly.

**Natural positive pairs exist (left/right organ, repeat scans, or longitudinal visits)**	Contrastive instance discrimination	iGWAS model for paired left-right retinal fundus images[36]	Biologically meaningful pairs can replace synthetic augmentation and preserve within-subject information.	The model reduces differences between paired views, which may remove biologically meaningful variation, for example, genetically influenced left-right anatomical asymmetry. Some contrastive objectives also require large batch sizes.
**A second imaging or physiological modality is available for the same individuals**	Cross-modal alignment (reconstruction plus contrastive learning)	Cross-modal autoencoder for cardiac MRI plus ECG[76]	A shared latent space can recover variants missed by either modality alone and permits cross-modal imputation.	Alignment may discard modality-specific variation; anatomical attribution is not straightforward.
**Genotypes are available during training, and maximum genetic enrichment is the priority**	Genotype-guided multimodal learning (not strictly image-only SSL)	ContIG[58]; Genetic InfoMax[48]	Genetic information is incorporated into the representation, potentially increasing downstream discovery power.	Circularity: the genotypes shaping the embedding are subsequently tested against it. SNP-h^2 and associations can be biased without strict sample separation.
**Images contain temporal dynamics (cine MRI or longitudinal image series)**	Spatiotemporal representation learning: 3D/4D VAEs, video transformers, or diffusion transformers	Emerging approaches: no direct peer-reviewed imaging-genetics GWAS examples identified.	Encodes motion, deformation, and strain instead of treating frames as unrelated static channels.	Interpretation approaches and replication standards are immature.

Abbreviations. AE, autoencoder; ECG, electrocardiogram; GWAS, genome-wide association study; IDP, imaging-derived phenotype; LV, left ventricle; MANOVA, multivariate analysis of variance; MOSTest, Multivariate Omnibus Statistical Test; MRI, magnetic resonance imaging; OCT, optical coherence tomography; SSL, self-supervised learning; UDIPs, Unsupervised Deep-Learning-Derived Imaging Phenotypes; VAE, variational autoencoder; VICReg, Variance-Invariance-Covariance Regularization.

This decision map is intended as practical guidance rather than a strict classification, as the optimal approach will also depend on cohort characteristics, computational resources, and validation data.

Reviewer #2 (Remarks to the Author):

I think the Authors have addressed the comments raised and the review reads better now.

Thank you for your feedback which helped to improve the manuscript.

Reviewer #4 (Remarks to the Author):

Thank you for the revised manuscript and detailed responses. I have reviewed the revised version and am satisfied that my previous concerns have been adequately addressed. I have no further major concerns and support acceptance.

Thank you for your feedback which helped to improve the manuscript.

Reviewer #5 (Remarks to the Author):

The manuscript has improved substantially in response to the previous reviews. The authors have clarified the relationship between self-supervised learning (SSL) and expert-defined imaging-derived phenotypes, expanded the historical and methodological context, simplified the tables, improved the figures, strengthened discussion of limitations, and removed AI-generated visual elements.

Overall, the authors have made a meaningful effort to address the previous comments, and the manuscript has clear potential. However, further revision is required to clarify the methodological scope, strengthen the critical synthesis, and ensure that claims about genetic discovery, reproducibility, and biological interpretation are appropriately calibrated. Some remaining comments are below:

1. Scope and terminology require further clarification. The manuscript uses “SSL” broadly, but some included examples appear to involve unsupervised learning, transfer learning, supervised models, or genotype-informed multimodal representation learning. The authors should either apply a stricter SSL definition or broaden the scope and clearly classify each approach.

Thank you for suggesting the need for clearer scope and terminology. In our previous revision, we defined our use of SSL and explained why autoencoders, although historically described as unsupervised models, are included as reconstruction-based SSL methods because the input image provides its own training target (introduction). We have retained this definition and made three targeted clarifications to further improve clarity.

First, at the beginning of the Self-Supervised Learning Approaches section, we now state:

Manuscript changes.

"Throughout this Review, SSL encompasses reconstruction-based and joint-embedding methods in which the supervisory signal is derived from the input data itself or from naturally paired views or modalities; this includes methods historically described as unsupervised, such as autoencoders, and hybrid studies when they contain an SSL component."

Second, we revised the description of TransferGWAS to clarify its hybrid design. The ADNI branch uses a VAE with a self-supervised image-reconstruction objective combined with supervised dementia prediction, whereas the ImageNet branch uses supervised pretraining followed by transfer to brain MRI. TransferGWAS is therefore included because it contains a reconstruction-based SSL component, while the supervised transfer-learning component is identified separately.

Manuscript changes.

"Another example is TransferGWAS[49] which derived phenotypes using two distinct strategies: a 3D CNN VAE trained on ADNI scans with a self-supervised image-reconstruction objective combined with supervised dementia prediction and a 2D ResNet-50 pretrained on ImageNet natural images via supervised transfer learning[80]."

Third, we now explicitly state in the multimodal-alignment subsection that genotype-informed methods such as ContIG and Genetic InfoMax are classified as multimodal SSL because matched imaging and genetic data from the same individual provide the supervisory signal without requiring manually annotated phenotype labels.

Manuscript changes.

"We classify genotype-informed methods such as ContIG and Genetic InfoMax as multimodal SSL because matched imaging and genetic data from the same individual serve as the supervisory signal without requiring manually annotated phenotype labels."

2. Potential circularity in genotype-informed embeddings needs explicit discussion. For approaches that use genotype data during representation learning and subsequently perform GWAS, the manuscript should discuss data leakage, sample overlap, and the need for non-overlapping training and association-testing datasets.

Thank you for your comment. We have now updated the text and table 2.

Manuscript changes.

"Multimodal alignment extends joint embedding across distinct input streams (Figure 2D). CLIP (Contrastive Language-Image Pre-Training) established the foundational framework by training paired image and text encoders with a contrastive objective[75], and the same paradigm has since been adapted to imaging-genetics, for example,

aligning retina images with genetics, or electrocardiogram (ECG) with cardiac MRI, into a shared latent space[58,76]. Genetic-guided approaches such as ContIG directly align an individual's image and genetic embeddings[58], in which case a separate GWAS data set is required to avoid label leakage. We classify genotype-informed methods such as ContIG and Genetic InfoMax as multimodal SSL because matched imaging and genetic data from the same individual serve as the supervisory signal without requiring manually annotated phenotype labels. When genotype data contribute to representation learning, using the same individuals for model training and subsequent GWAS can create circularity and information leakage because the tested genotypes have already influenced the phenotype definition. Representation-learning and GWAS cohorts should therefore be non-overlapping, and any sample overlap should be reported explicitly. The simplest alignment objective minimizes squared distance between paired embeddings: $L_{align} = \| z_{img} - z_{gen} \|^2$. In plain terms, the loss is small when imaging and genetic embeddings of the same individual sit close together in the shared latent space, so the two modality-specific encoders are jointly trained to agree on a common representation per person (Figure 2D). In practice, contrastive or auxiliary losses are often needed to preserve individual-level variation and avoid representational collapse."

"Table 2. GWAS-relevant trade-offs: For genotype-informed methods such as ContIG and Genetic InfoMax, using genetics in the training objective creates potential circularity if the same individuals (or related pairs) are included in both model training and subsequent GWAS. Non-overlapping representation-learning and GWAS samples are therefore needed to avoid inflated or difficult-to-interpret association and heritability estimates. Cross-modal approaches also require paired data from the same individuals."

3. The comparison between expert-defined IDPs and SSL-derived phenotypes remains too categorical. Table 1 is useful but should better acknowledge that both approaches depend on preprocessing, technical choices, and cohort characteristics. SSL approaches do not necessarily operate without anatomical assumptions, and expert-defined phenotypes are not uniformly more reproducible.

Thank you for your comment. We have now revised Table 1 to clarify that both expert-defined IDPs and SSL-derived phenotypes depend on preprocessing, technical choices, and cohort characteristics. We removed the implication that SSL is free of anatomical assumptions, moderated the reproducibility comparison, and added new rows addressing susceptibility to technical and non-biological variation and GWAS multiple-testing burden.

Manuscript changes. Miscellaneous changes in Table 1.

4. The review would benefit from stronger critical synthesis. Rather than primarily listing architectures and applications, the manuscript should more directly address when SSL-derived features provide information beyond conventional IDPs, how this should be evaluated, and what evidence is needed for biological or clinical interpretation.

Thank you for this important suggestion. We agree that the value of SSL-derived phenotypes should not be assessed primarily by the architecture used or by locus counts alone. We have added a concise paragraph at the end of the SSL-Based Imaging Genetics Framework section, before the discussion of specific SSL approaches. The new text explains that SSL-derived phenotypes may be particularly complementary when relevant variation is distributed across an organ or involves complex shape, texture, spatial relationships, or temporal dynamics that are not adequately summarized by predefined measurements.

Manuscript changes.

"Evaluating the Added Value of SSL-Derived Phenotypes Beyond Conventional IDPs

SSL-derived phenotypes are most likely to provide information beyond conventional IDPs when relevant imaging variation is distributed across an organ or involves complex shape, texture, spatial relationships, or temporal dynamics that cannot be summarized adequately by a small set of predefined measurements. However, added value should not be inferred solely from the number of genome-wide significant loci, because locus counts may also reflect differences in phenotype dimensionality, sample size, statistical power, or definitions of novelty. Ideally, SSL-derived phenotypes and conventional IDPs should be compared in the same participants under similar analytical conditions, with complementarity shown by reproducible genetic associations that persist after accounting for established IDPs. Biological or clinical interpretation requires additional evidence such as feature stability, independent genetic replication, functional support, or associations with meaningful clinical traits and outcomes."

5. Interpretability and biological validation should be discussed more rigorously. Heritability, saliency maps, or association signals alone do not establish that a latent feature is a biologically meaningful phenotype. The authors should distinguish image localization, feature stability, genetic replication, biological mechanism, and clinical relevance.

Thank you for this important point. We agree that heritability, saliency maps, or a significant association do not by themselves establish that a latent feature is a biologically meaningful phenotype. We have expanded the Methodological and Interpretability Barriers subsection with a new paragraph that distinguishes five separate levels of evidence.

Manuscript changes.

"**Methodological and Interpretability Barriers** For SSL-specific challenges, domain shift poses a particularly acute barrier for self-supervised models, which are often sensitive to acquisition artifacts. Models trained on uniformly collected and processed UKBB data may fail when applied to diverse and heterogeneous clinical scans. A related concern is that SSL models may inadvertently encode non-biological variation such as scanner differences, acquisition artifacts, or ancestry-related morphological differences as latent features, potentially confounding downstream GWAS. Current studies mitigate this by including standard covariates (e.g., age, sex, genetic principal components) in association models, but systematic evaluation of residual bias in learned representations remains limited. Furthermore, despite the self-supervised label, most SSL pipelines still rely on conventional preprocessing such as skull stripping and registration which introduces its own variability. Computational burden is another significant constraint. Training models on high-dimensional modalities (e.g., 4D cardiac cine MRI) is resource-intensive. This creates a barrier for smaller and less-well-resourced research groups and raises concerns about the carbon footprint of model development. Simultaneously, the high dimensionality of latent spaces can severely dilute statistical power. While dimensionality reduction (e.g., PCA) helps, there is no consensus on the optimal balance between retaining morphological detail and maintaining statistical efficiency.

Interpretability and biological validation are not a single step. They involve five separate questions, and each needs its own evidence. First, image localization asks which image regions contribute to a latent feature. Saliency maps, occlusion analyses, and decoder-based perturbations answer this, but knowing where a feature comes from does not tell us what it means[37,59]. Second, feature stability asks whether the same feature appears again when the model is retrained or when preprocessing, scanners, or cohorts change. This is difficult because latent dimensions are not ordered in a fixed way, so a dimension from one model does not correspond to the same dimension in another[87,88]. Third, genetic replication asks whether an association holds in an independent sample. It shows the association is real, but not what the phenotype measures. Replication in a held-out split of the same cohort is also weaker than replication in a separate cohort[89]. Fourth, biological mechanisms require further evidence, such as fine-mapping, colocalization with molecular quantitative-trait loci, pathway analyses, or experimental follow-up[90,91]. Fifth, clinical relevance requires association with meaningful traits or outcomes, added value beyond established IDPs, and validation in independent clinical cohorts[92–94]. Most current SSL imaging-genetics studies address only some of them. A latent feature is therefore better described by the specific evidence available for it than by a general label such as "biologically validated."

Interpretability remains a persistent difficulty even at the first of these levels. While perturbation-based decoding and saliency mapping provide partial insights, they have concrete limitations: decoder perturbations often smear effects across anatomical boundaries rather than being localized, and saliency maps can saturate, obscuring the magnitude of specific features [37,95]. Feature stability is rarely tested directly. Ensembling across many independently trained models, as in the Unsupervised Phenotype Ensemble, reduces the effect of single-model instability but does not measure it[46]. Clinical translation from SSL-discovered loci to therapeutic targets also remains largely theoretical, with experimental follow-up of SSL-GWAS loci essentially absent from the literature."

6. The literature-search strategy should be more transparent. Even as a narrative review, the manuscript should provide the search date, databases, representative search terms, inclusion/exclusion criteria, and treatment of preprints. The literature should be updated and bibliographic details checked. Recent relevant studies should be included where appropriate, and preprints should be clearly labelled or replaced by peer-reviewed publications when available.

Thank you for this suggestion. We expanded the literature-search description to include the search date, databases, representative search terms, inclusion and exclusion criteria, and treatment of preprints. We have now revisited the bibliography, added some relevant citations, and updated the preprints to published manuscripts.

Manuscript changes

"We searched PubMed and Google Scholar through May 2026 and screened the reference lists of included studies. Representative terms were combined: ("self-supervised learning" OR "unsupervised representation learning" OR autoencoder OR "contrastive learning") AND ("medical imaging" OR MRI OR cardiac imaging OR retinal imaging OR fundus OR OCT) AND ("genome-wide association study" OR GWAS OR "genetic association"). We included studies that applied SSL or unsupervised representation learning directly to medical images and used the learned features as phenotypes for genome-wide association testing. We excluded studies that were based only on predefined IDPs, used supervised models without an SSL component, or did not include GWAS."

7. The conclusions should be moderated. The evidence base remains relatively small, predominantly based on UK Biobank data, and has limited cross-cohort replication or functional validation. The conclusion should present SSL-derived phenotypes as promising complementary tools rather than established, reproducible, or clinically actionable biomarkers.

Thank you for your suggestion. We have now updated the conclusion by adding your suggested line

Manuscript changes. “Important challenges remain, including limited interpretability, lack of standardized pipelines, high computational demands, and restricted reproducibility and generalizability beyond largely European ancestry cohorts. SSL-derived phenotypes are therefore best regarded at present as promising complementary tools rather than established, clinically actionable biomarkers.”
